# Humanized Antibodies Targeting Ectodomains of IL‐6R and GP130 Suppress IL‐6/STAT3 Signalling and Tumour Growth in Breast Cancer Models

**DOI:** 10.1111/jcmm.71250

**Published:** 2026-06-18

**Authors:** Satyajit Dey Pereira, Guruprasad Baipadithaya, Keshava Prasad, Ritam Naha, Lavanya Prakash Acharya, Ganesh Prasad Uppenda Gopalakishna, Ramyaa Periasamy, Arun Chandrashekar, Shama Bhat, Manjunath B. Joshi, Kapaettu Satyamoorthy

**Affiliations:** ^1^ Department of Cell and Molecular Biology, Manipal School of Life Sciences Manipal Academy of Higher Education Manipal India; ^2^ Bhat Bio‐Tech India (P) Ltd. Bengaluru India; ^3^ Department of Ageing Research, Manipal School of Life Sciences Manipal Academy of Higher Education Manipal India; ^4^ SDM Centre for Cellular and Molecular Sciences, SDM College of Medical Sciences and Hospital Shri Dharmasthala Manjunatheshwara (SDM) University Dharwad India

**Keywords:** breast cancer, GP130, humanized monoclonal antibodies, IL‐6, IL‐6R

## Abstract

Interleukin‐6 (IL‐6) signalling is a key driver of breast cancer progression, activating pro‐survival, pro‐inflammatory and metastatic programs through its receptors, IL‐6R and GP130. Elevated IL‐6 levels correlate with poor prognosis in breast cancer patients and persistent IL‐6/STAT3 activation promotes tumour proliferation, metastasis, angiogenesis and therapy resistance. Although therapeutics targeting this pathway have shown promise, most have focused on individual signalling nodes and yielded limited efficacy in breast tumours. In this study, we generated murine monoclonal antibodies targeting ectodomains of IL‐6R and GP130 receptors involved in IL‐6/IL‐6R/GP130 complex formation and humanized the lead candidates E17 (anti–IL‐6R) and NA7 (anti–GP130). The resulting humanized antibodies, huE17 and huNA7, exhibited target specificity and effectively inhibited IL‐6 induced STAT3 activation and tumour promoting phenotypes in vitro. In vivo evaluation using orthotopic breast cancer xenografts and agarose plug angiogenesis assays showed both individual and combined antibody treatments significantly suppressing tumour growth. Structural modelling suggested that huE17 and huNA7 engage receptor surfaces spanning multiple ectodomains of IL‐6R and GP130, consistent with disruption of IL‐6/IL‐6R/GP130 complex receptor assembly. Together, these findings support the preclinical development of huE17 and huNA7 and the potential of dual IL‐6R and GP130 targeting in IL‐6–driven breast cancer.

**Abbreviations**
CDR: Complementarity Determining Region
GP130: Glycoprotein 130
IL‐6: Interleukin‐6
IL‐6R: Interleukin‐6 Receptor
JAK: Janus Kinase
LCAT: Lecithin cholesterol Acyltransferase
mIL‐6R: membrane bound Interleukin‐6 Receptor
pSTAT3: Phosphorylated Signal transducer and activator of transcription 3
sGP130: soluble Glycoprotein 130
sIL‐6R: soluble Interleukin‐6 Receptor
STAT3: Signal transducer and activator of transcription 3

## 1 Introduction

Breast cancer is the second most commonly diagnosed cancer worldwide, with an estimated 2.3 million new cases and 660,000 deaths in 2022, accounting for 15.4% of cancer‐related deaths in women [1, 2]. Breast cancers are classified into luminal A, luminal B, HER2‐enriched, basal‐like, and claudin‐low subtypes based on the expression status of cell surface oestrogen receptor (ER), progesterone receptor (PR), and human epidermal growth factor receptor 2 (HER2) [3]. Despite multiple available treatment modalities, breast cancer remains a formidable clinical challenge due to its complex aetiology, metastatic relapse, tumour heterogeneity and therapy resistance [4].

Interleukin‐6 (IL‐6) is a pleiotropic cytokine secreted by immune, stromal and tumour cells [5, 6]. IL‐6 signalling occurs through a receptor complex comprising IL‐6R (Interleukin‐6 receptor) and GP130 (Glycoprotein 130). IL‐6R exists in both transmembrane (mIL‐6R) and soluble forms (sIL‐6R) [7]. Binding of IL‐6 to mIL‐6R initiates classical signalling whereas binding to sIL‐6R activates trans‐signalling; both modes induce GP130 homodimerization [8, 9]. In addition, IL‐6 cluster signalling or trans‐presentation has been recently described where a membrane bound IL‐6/IL‐6R complex can activate GP130 signalling on neighbouring cells [10, 11]. These mechanisms trigger Janus kinase (JAK) mediated phosphorylation and culminate in STAT3 (Signal transducer and activator of transcription 3) phosphorylation, dimerization and activation. Activated STAT3 drives transcription of genes supporting inflammation, proliferation, epithelial‐mesenchymal transition, apoptosis inhibition, immune evasion, therapy resistance, angiogenesis and tumour stemness in several cancers [12, 13, 14, 15]. Constitutive STAT3 activation is observed in over 50% of all breast cancers, and is frequently driven by aberrant IL‐6 signalling [16]. Sustained IL‐6 secretion results in a constitutive IL‐6/STAT3 autocrine loop wherein IL‐6 activates STAT3 and STAT3 in turn transcriptionally induces IL‐6 expression [17].

Dysregulated IL‐6 expression, elevated serum and tissue levels have been reported across multiple malignancies [18, 19, 20, 21, 22]. In breast cancer increased IL‐6 levels are associated with disease progression and poor prognosis [23, 24, 25]. The major breast cancer subtypes rely on IL‐6 signalling to varying degrees. In ER‐positive tumours that typically respond to endocrine therapy, IL‐6 signalling can suppress ER receptor expression and drive endocrine resistance, metastasis and tumour recurrence [26, 27]. In HER2 positive tumours—elevated IL‐6 and phosphorylated STAT3 (pSTAT3) levels contribute to resistance to HER2 targeting drugs like Trastuzumab [28]. Triple negative breast cancers exhibit highest IL‐6 and pSTAT3 levels and blockade of this cascade significantly inhibits tumour growth [29]. These observations highlight the IL‐6/IL‐6R/GP130 axis as a critical mediator of breast cancer progression and a potential therapeutic target [29, 30].

Several therapeutic approaches targeting the IL‐6 signalling pathway including monoclonal antibodies against—IL‐6 (siltuximab and sirukumab), IL‐6R (tocilizumab and sarilumab) and small‐molecule inhibitors of STAT3 have demonstrated anti‐tumour efficacy in preclinical settings and are being clinically evaluated [31]. In parallel, GP130 has also been explored as a therapeutic target through antibodies for inflammatory conditions [32], repurposing of oestrogen receptor modulating drugs presently available in the market—raloxifene and bazedoxifene [33, 34] for cancer therapy and soluble GP130 Fc fragment molecules (sGP130) being trialled for chronic inflammatory conditions [35]. However, no IL‐6 pathway targeting therapy is currently approved for breast cancer, underscoring the complexity of targeting this axis and the challenges associated with achieving effective disruption of IL‐6 signalling.

Humanized monoclonal antibodies offer a clinically relevant strategy to selectively disrupt cytokine receptor complex components with high specificity and reduced immunogenicity [36]. In this study, we report the development of humanized neutralizing monoclonal antibodies directed against IL‐6R and GP130, followed by evaluation of their ability to disrupt the IL‐6/IL‐6R/GP130 signalling and its downstream oncogenic consequences through breast cancer models. The effects of IL‐6R and GP130 blockade were evaluated individually and in combination in vivo. While individual targeting of IL‐6R or GP130 has been explored previously, concurrent IL‐6R and GP130 blockade in breast cancer models has not been widely reported. Our findings establish the preclinical activity of humanized antibodies targeting distinct components of the IL‐6 receptor complex and assess the feasibility of dual receptor targeting and its impact on IL‐6/STAT3‐driven tumour growth in vivo.

## 2 Materials and Methods

### 2.1 Cell Culture

CHO, HEK293, MCF‐7, MDA‐MB‐231 and MCF‐10A cell lines were procured from ATCC and cultured according to supplier's protocol. HUVEC cells were isolated from umbilical veins and cultured as described previously [37] with approval from the Institutional Ethics Committee, Kasturba Medical College. All cell lines were maintained at 37°C in 5% CO_2_. Cancer cell lines were authenticated through STR profiling.

### 2.2 In Silico Expression Analysis

mRNA expression levels of IL‐6R, GP130 and IL‐6 across breast cancer stages and corresponding matched normals as well as in molecular subtypes and normal tissue were analysed by accessing TCGA RNA‐seq data [38] via UCSC Xena browser [39]. Expression pattern in breast cancer and non‐tumorigenic cell lines was assessed using Genevestigator [40]. Expression levels of IL‐6 in the breast tumour microenvironment was profiled in publicly available single cell sequencing datasets using TISCH2 [41].

### 2.3 Identification of Interacting Domains and Amino Acids

IL‐6R and GP130 receptor protein domains and post‐translational modifications were accessed from UniProt entries (IL‐6R UniProt ID: P08887; GP130 UniProt ID: P40189). Amino acid residues involved in the IL‐6/IL‐6R/GP130 complex (PDB ID: 1P9M) were visualized using the open‐source PyMOL molecular graphics system.

### 2.4 Cloning of IL‐6R and GP130 Ectodomains

Ectodomains of IL‐6R and GP130 were cloned into pOptiVEC‐TOPO vector (Invitrogen, USA) and transfected into CHO cells with stable clones selected using 7.5 μM methotrexate (Sigma‐Aldrich, USA). Recombinant proteins were purified from culture supernatants by Ni‐NTA affinity column chromatography (Merck Millipore, USA) and validated by Mass Spectrometry.

### 2.5 Hybridoma Development

Hybridoma development was carried out at Bhat Bio‐Tech India (P) Ltd. (CPSEA No. 1350/C/10/CPCSEA). All procedures were performed following Institutional Animal Ethics Committee approval (IAEC) (BBI/IAEC/JULY/2017/01 and BBI/IAEC/JULY/2018/02). 8‐ to 10‐week‐old female BALB/c mice were immunized with purified recombinant IL‐6R or GP130 protein along with complete Freund's adjuvant and then with a booster dose of incomplete Freund's adjuvant after 3 to 4 weeks. Immunization was continued until sufficient antibody levels in serum were detected by ELISA (Abcam, USA). The mice were euthanized by cervical dislocation, and hybridomas were generated as previously described [42]. All hybridomas were screened by ELISA (Abcam, USA), isotyped with an isotyping kit (Sigma‐Aldrich, USA) and purified using Protein A/G agarose beads (Santa Cruz, USA). Figure S1 provides an overview of the cloning and hybridoma development strategy.

### 2.6 Cytotoxicity Assessment

Effects of the murine E17 anti‐IL‐6R and NA7 anti‐GP130 and humanized huE17 and huNA7 antibodies on cell viability were assessed in MCF‐7 and MDA‐MB‐231 breast cancer cells by CCK8 assay (Dojindo, Japan). The cells were serum starved with 1% FBS for 24 h and then treated with different concentrations of antibodies for 48 h. Absorbance was measured at 450 nm. IC_50_ values were calculated for each antibody.

### 2.7 LCAT and STAT3 Reporter Assays

The Lecithin‐ Cholesterol Acyl Transferase (LCAT) promoter which harbours a STAT3 binding site was cloned in pGL3 plasmid (Promega, USA) as described previously [43]. STAT3 response repeat elements were also cloned into pGL3‐TK‐Luc luciferase reporter plasmid as described in the Methods S1. Reporter assays were conducted by transfecting the constructs into MCF‐7/MDA‐MB‐231 cells using Lipofectamine (Invitrogen, USA). Renilla luciferase construct (Promega, USA) was used as the internal standard. Cells were treated with IL‐6 (25 ng/mL) (Invitrogen, USA) in the presence/absence of murine or humanized antibodies (100 ng/mL). Luciferase activity was measured after 48 h using the Dual Luciferase Reporter assay kit (Promega, USA).

### 2.8 In Vitro Angiogenesis Assay

Matrigel‐based HUVEC tube formation assay was performed to evaluate effects of the mouse E17 anti‐IL‐6R and NA7 anti‐GP130 mouse antibodies on IL‐6 induced endothelial tube formation. 20,000 HUVEC cells were seeded in each well of a 96‐well plate coated with matrigel and the following groups were maintained—control, IL‐6 (25 ng/mL), E17 (100 ng/mL), NA7 (100 ng/mL), IL‐6 (25 ng/mL) + E17 (100 ng/mL), IL‐6 (25 ng/mL) + NA7 (100 ng/mL). Basic Fibroblast Growth Factor (bFGF) (10 ng/mL) (Invitrogen, USA) was used as positive control. Cells were incubated for 6 h after which the tubes formed by cells were observed under the microscope, counted and photographed.

### 2.9 Specificity of IL‐6R and GP130 Antibody Clones

Specificity of the murine (E17, NA7) and humanized (huE17 and huNA7) antibodies for the IL‐6R/GP130 proteins in breast non‐tumourigenic and cancer cell lysates was assessed through western blotting of cell line lysates onto methanol‐activated PVDF membrane (Bio‐Rad, USA) post SDS‐PAGE. The membranes were blocked with 5% BSA (bovine serum albumin) in TBST (Tris‐Buffered Saline with Tween‐20), probed with mouse/humanized antibodies (1 μg each) overnight at 4°C and subsequently incubated with goat anti‐mouse/anti‐human IgG HRP secondary antibodies (Abcam, USA) and then visualized using ECL western blotting substrate reagent (Biorad, USA) and images captured using ImageQuant LAS 4000 (GE HealthCare, USA). The IL‐6R blot was thereafter stripped using mild stripping buffer and re‐probed for actin.

### 2.10 STAT3 Phosphorylation Neutralization Assessment by Western Blotting

MCF‐7 and MDA‐MB‐231 cells were treated with IL‐6 (25 ng/mL) for 1 h in the presence or absence of purified mouse/humanized E17 and NA7 antibodies (100 ng/mL). 30 μg of the isolated cell lysates were resolved by SDS‐PAGE, transferred onto a PVDF membrane (Biorad, USA), blocked with 5% BSA in TBST, and probed with β‐actin (Invitrogen, USA) and Phospho‐STAT3^**Tyr705**^ (CST, USA) antibodies. Goat anti‐mouse IgG HRP secondary antibodies (Abcam, USA) were used for secondary staining. Proteins were visualized using ECL western blotting substrate reagent (Biorad, USA), and images captured using ImageQuant LAS 4000 (GE HealthCare, USA). The phospho‐STAT3 blots were stripped using mild stripping buffer and then re‐probed for Total STAT3.

### 2.11 Humanization of Mouse E17 Anti‐IL‐6R and NA7 Anti‐GP130 Antibodies

VH and VL regions of the mouse E17/NA7 antibody producing murine IgG2b hybridoma cells were amplified from hybridoma cDNA and sequenced using different light chain and heavy chain degenerate primer combinations as previously described [44]. Humanization was performed by CDR grafting [45] using IMGT/VQUEST and TABHU platforms [46, 47] to identify humanized VH and VL sequences which were cloned into pVITRO1‐M80‐F2‐IgG1/κ vectors (Invivogen, USA) [48]. These plasmids were transfected into HEK293 cells under hygromycin selection (20 μg/mL) (HiMedia, India). Detailed methodology for antibody humanization is provided in Methods S1.

### 2.12 Molecular Modelling and Simulation of HuE17 Anti‐IL‐6R and HuNA7 Anti‐GP130 Interaction With IL‐6R and GP130 Receptors

ColabFold, an AlphaFold2‐based protein structure prediction framework, was used to predict the 3D structure of the huE17 and huNA7 antibodies from the full‐length heavy and light chain amino acid sequences [49]. To specifically identify the antigen‐binding regions of the antibodies, the variable fragment (Fv) structures of huE17 and huNA7 were predicted using the ABodyBuilder3 (ABB3) tool on the SABPred server [50]. The antibody mode of the ClusPro server [51] was used to dock the interactions between the predicted Fv structures of the huE17/huNA7 antibodies and IL‐6R and GP130 receptors, respectively. Putative epitopes were defined as receptor residues located within 4 Å of the antibody Fv in the highest‐ranked docking complexes. The highest‐ranked antibody–receptor complexes predicted by the ClusPro server were selected for computational binding affinity estimation using CSM‐AB [52].

### 2.13 Western Blot to Confirm Antibody Humanization

E17‐IL‐6R and NA7‐GP130 pVITRO vector transfected Hek293 cell lysates and Protein A resin affinity chromatography purified huE17 and huNA7 antibodies were resolved in a 12% SDS‐PAGE Gel, blotted onto a methanol activated PVDF membrane, blocked with 5% BSA in TBST for 1 h and then probed with anti‐human secondary antibody (Abcam, USA) for 1 h. Proteins were visualized using ECL western substrate reagent (Biorad, USA) and images captured using ImageQuant LAS 4000 (GE HealthCare, USA).

### 2.14 Basement Membrane Invasion Assay

Basement membrane invasion assay was performed to quantify the ability of MCF‐7 and MDA‐MB‐231 cells to cross a barrier consisting of basement membrane extract and an 8 μM polyester filter under the influence of IL‐6 using cultrex BME Cell Invasion Assay kit (Trevigen, USA). 50,000 MCF‐7/MDA‐MB‐231 cells were seeded per well, after overnight serum starvation and were treated with different combinations of IL‐6 (25 ng/mL) (chemoattractant) and huE17 anti‐IL‐6R and huNA7 anti‐GP130 antibodies (1 μg/mL). Non‐Immuno IgG (1 μg/mL) was used as control for the antibodies. The ability of the cells to cross the basement membrane extract coated 8 μM polyester filter was quantified as cell invasion % with the help of a standard curve generated as per the kit manufacturers protocol.

### 2.15 Agarose Plug Assay to Assess Effect of HuE17 Anti‐IL‐6R and HuNA7 Anti‐GP130 on IL‐6 Induced Angiogenesis In Vivo

In vivo angiogenesis studies were conducted at Central Animal Research Facility (CARF), Manipal (CPCSEA no: 94/PO/ReBi/S/1999/CPCSEA) after obtaining ethical clearance from the Institutional Animal Ethical Committee (IAEC) at Kasturba Medical College, Manipal Academy of Higher Education (IAEC/KMC/48/2019). Swiss albino mice (6 to 8 weeks) were randomized into 6 groups (*n* = 5/group) and treated with IL‐6 (250 ng/kg), IL‐6 + huE17(1 mg/kg), IL‐6 + huNA7(1 mg/kg), huE17 only (1 mg/kg), huNA7 (1 mg/kg) only, non‐Immuno IgG (negative control) (1 mg/kg) and untreated control. Treatments were mixed in 2% agarose to form solidified plugs. Under ketamine (75 mg/kg) and xylazine (5 mg/kg) anaesthesia, plugs were implanted subcutaneously through a dorsal incision and the wound was sutured. After 7 days, mice were euthanized by cervical dislocation, and plug sites were excised, fixed in 4% formalin, sectioned, and stained with haematoxylin and eosin for histological assessment of neovascularization. All surgeries were performed by the same animal handler and on the same day. Further animal cages were positioned on the same rack and daily checks were conducted to ensure ample water and food were available. These measures were taken to reduce the role of external/confounding factors.

### 2.16 In Vivo Efficacy Testing of HuE17 Anti‐IL‐6R and HuNA7 Anti‐GP130 Antibodies in Nude Mouse Orthotopic Breast Tumour Xenograft Models

All in vivo efficacy studies were conducted at CARF, Manipal under the same ethical clearance as stated for the in vivo angiogenesis assay. 7.5 × 10^6^ cells MDA‐MB‐ 231 cells were suspended in a mixture of PBS: FBS: Matrigel in a 1:1:2 ratio and  injected subcutaneously into mammary fat pad of 5‐ to 7‐week‐old athymic BALB/C nude mice to induce breast tumours. 72‐h post injection mice were randomized into four different treatment groups (*n* = 4/group)—untreated control, huE17 antibody (0.5 mg/kg bodyweight), huNA7 antibody (0.5 mg/kg bodyweight), huE17 + huNA7 (0.25 mg/kg bodyweight of each antibody) and humanized antibodies were administered weekly via intraperitoneal injection for 4 weeks. Tumour volume was measured weekly using vernier callipers. One week after treatment completion, mice were sacrificed, tumours excised, photographed, and weighed. Tumour sections were subjected to IHC staining for CD31 (CST, USA) and Vimentin (Novus, USA) to evaluate angiogenesis and EMT. All injections and treatments were performed by the same handler under identical conditions to minimize variability.

### 2.17 Statistical Analysis

All experiments were performed in biological replicates, and the number of independent experiments is indicated in the corresponding figure legends. Statistical analyses were performed using Graphpad Prism 8.0 software. The data are represented as mean ± standard deviation (SD). The statistical tests used for each experiment are indicated in the corresponding figure legends. Statistical significance was defined as *p ≤ 0.05, **p ≤ 0.01, ***p ≤ 0.001 and ****p ≤ 0.0001.

## 3 Results

### 3.1 Expression Profiling and Target Domain Mapping of IL‐6, IL‐6R and GP130

**FIGURE 1:**
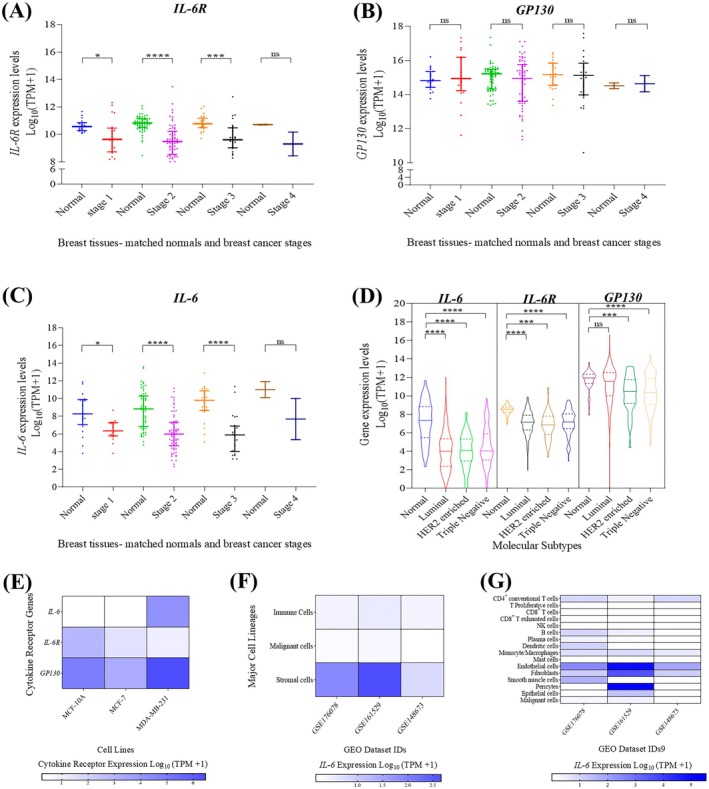
Expression profiles of IL‐6, IL‐6R and GP130: (A) IL‐6R (B) GP130 and (C) IL‐6 expression across TCGA‐breast cancer stages with matched normal tissue (stage 1 *n* = 20, stage 2 *n* = 61, stage 3 *n* = 24, stage 4 *n* = 2). (D) Expression levels in normal tissue (*n* = 58) and molecular subtypes (Luminal *n* = 454, HER2‐enriched *n* = 31, triple negative *n* = 113). Statistical significance for panels A‐D was determined using one‐way ANOVA with Sidak's multiple comparisons test (*p ≤ 0.05, **p ≤ 0.01, ***p ≤ 0.001 and ****p ≤ 0.0001). (E) Genevestigator based expression in non‐tumorigenic breast epithelial and breast cancer cell lines. (F) IL‐6 expression within breast tumour microenvironment (TISCH 2) (G) IL‐6 expression across stromal, immune and malignant cells.

In silico analysis revealed lower IL‐6R expression levels in different breast cancer stages (Figure 1A) and molecular subtypes (Figure 1D) in comparison to stagewise matched normals and normal tissue respectively, although absolute Log10(TPM + 1) values indicated retained receptor expression. In the case of cell lines IL‐6R showed high expression in the non‐tumorigenic MCF‐10A compared to the breast cancer cell lines (Figure 1E). In contrast, GP130 showed uniformly high expression levels across tumour stages (Figure 1B), molecular subtypes (Figure 1D) and cell lines (Figure 1E). IL‐6 expression was lower in different breast tumour stages (Figure 1C) and subtypes (Figure 1D) compared to matched normals. Just as in the case of IL‐6R, while expression levels were lower than in normal tissue, the Log_10_(TPM + 1) levels were still relatively high. Among cell lines MDA‐MB‐231 exhibited elevated expression (Figure 1E). Given persistent reports of elevated circulating IL‐6 in patients [18], we assessed the possibility of paracrine action of tumour microenvironment derived IL‐6 in breast tumours. Single‐cell RNA‐seq analysis of breast tumour microenvironment datasets revealed stromal‐derived IL‐6 production, with endothelial cells, fibroblasts, and pericytes representing major contributors in multiple datasets (Figure 1F,G). These observations, along with the reports of elevated circulating IL‐6 and soluble IL‐6R levels [53] in breast cancer patients, support the rationale of targeting the IL‐6 signalling cascade despite heterogeneous expression patterns.

Structural mapping and molecular modelling identified IL‐6R domains II and III (P113–M311) and GP130 domains I–III (D26–Y321) (Figure S2A,B), and the key amino acid residues involved in the IL‐6/IL‐6R/GP130 complex (Figure S3A,B). Recombinant ectodomains were successfully purified and validated by SDS‐PAGE and mass spectrometry (Figures S4 and S5) and used for hybridoma generation.

### 3.2 E17 Anti‐IL‐6R and NA7 Anti‐GP130 Mouse Monoclonal Antibody Clones Exhibit Effects on Breast Cancer Cell Viability and Inhibit IL‐6 Signalling

**FIGURE 2:**
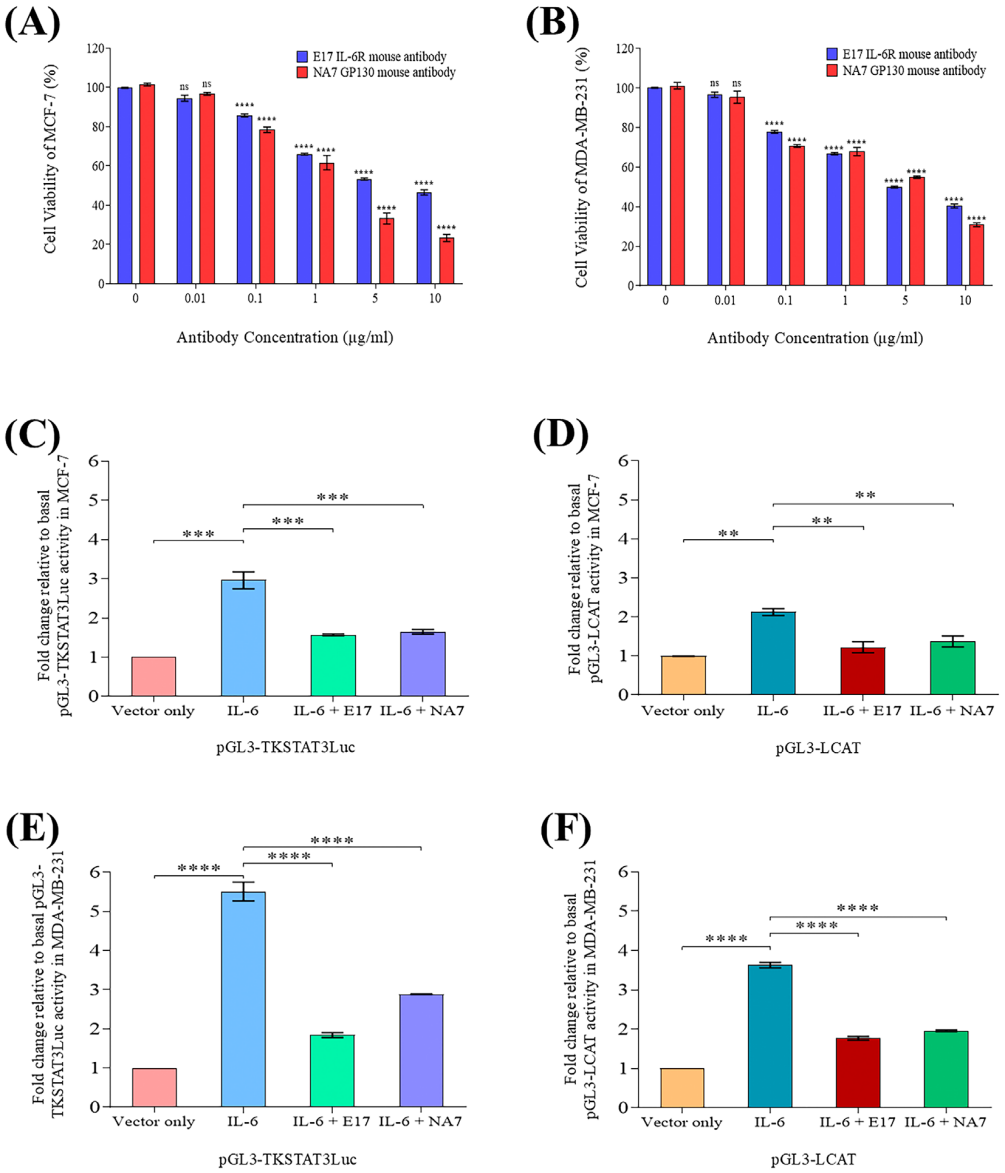
Screening of candidate mouse monoclonal antibodies against IL‐6R and GP130: Cytotoxicity of E17 and NA7 against IL‐6R and GP130 respectively in (A) MCF‐7 and (B) MDA‐MB‐231 cells. Statistical significance for panels A and B was determined by comparison of percentage cell viability at each treated concentration relative to untreated controls using two‐way ANOVA with Dunnett's multiple comparisons test (*p ≤ 0.05, **p ≤ 0.01, ***p ≤ 0.001 and ****p ≤ 0.0001). (C) pGL3‐TKSTAT3Luc response elements luciferase reporter assay and (D) pGL3‐LCAT promoter luciferase reporter assay in MCF‐7 cells. (E) pGL3‐TKSTAT3Luc response elements luciferase reporter vector assay and (F) pGL3‐LCAT promoter luciferase reporter assay in MDA‐MB‐231 cells. Data shown as fold change versus basal activity. Statistical significance for panels C‐F was determined using one‐way ANOVA with Sidak's multiple comparisons test (****p ≤ 0.0001, ***p ≤ 0.001, **p ≤ 0.01, *p ≤ 0.05). Data are represented as mean ± SD (*n* = 3).

Among the screened clones, results for only the two best performing clones, the E17 anti‐IL‐6R and NA7 anti‐GP130 mouse antibodies are presented. The IC_50_ values in the MCF‐7 cell line for the E17 and NA7 mouse monoclonal antibodies derived from the cell viability assays were approximately 5 μg/mL and between 2.5 to 3 μg/mL respectively (Figure 2A). In the case of the MDA‐MB‐231 cells the IC₅₀ value lay in the range of 4.5 to 5 μg/mL for both clones. (Figure 2B). IL‐6 stimulation induced a 2.5 to 3‐fold and 5 to 5.5‐fold rise in luciferase activity downstream of STAT3 response elements in MCF‐7 and MDA‐MB‐231 cells respectively (Figure 2C,E respectively) which was significantly inhibited by both E17 and NA7 antibodies. Similarly, in the LCAT promoter luciferase reporter assay IL‐6 induced a 2‐fold and 3 to 3.5‐fold increase in luciferase activity which was significantly suppressed by both antibodies (Figure 2D,F) confirming functional neutralization of IL‐6 signalling.

### 3.3 E17 and NA7 Mouse Monoclonal Antibodies Modulate IL‐6 Induced STAT3 Phosphorylation and Endothelial Tube Formation In Vitro

**FIGURE 3:**
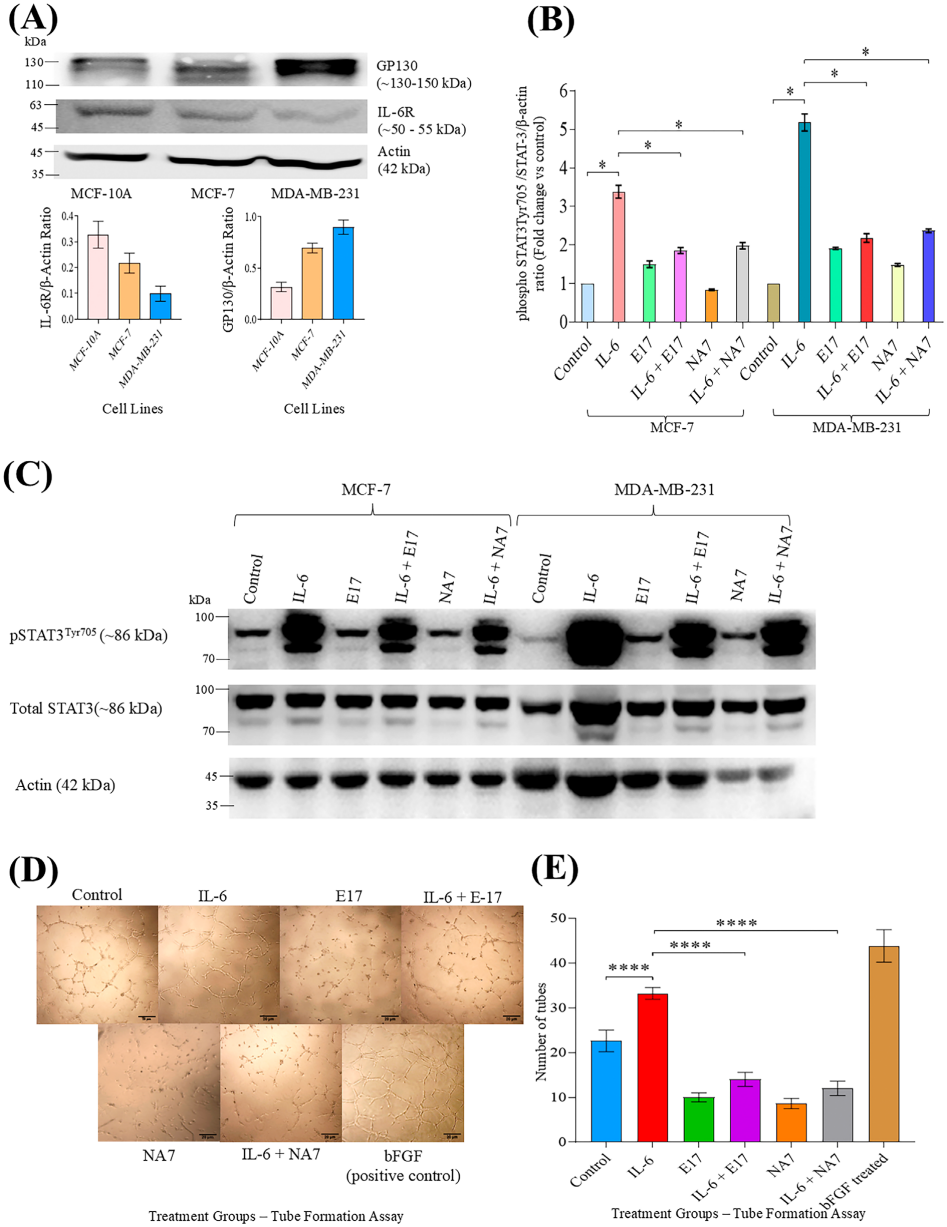
Functional characterization of E17 and NA7 mouse monoclonal antibodies: (A) Binding specificity of E17 and NA7 mouse antibodies for IL‐6R and GP130 with densitometric quantification. (B, C) Densitometric quantification and representative western blots of effects of mouse antibodies on IL‐6 induced STAT3 phosphorylation. pSTAT3 levels were normalized to Total STAT3 and β‐Actin levels and represented as fold change versus control. Statistical significance for indicated pairwise comparisons was determined by two‐tailed unpaired *t*‐test with Welch's correction (*p ≤ 0.05, **p ≤ 0.01, ***p ≤ 0.001 and ****p ≤ 0.0001). (D) Representative images for IL‐6 induced endothelial tube formation under mouse antibody treatment, Scale bars = 20 μm. (E) Quantification of the average number of tubes counted (in five fields) formed after 6 h of treatment. Statistical significance was determined using one‐way ANOVA with Sidak's multiple comparisons test (*p ≤ 0.05, **p ≤ 0.01, ***p ≤ 0.001 and ****p ≤ 0.0001). Representative western blots and tube formation images are shown from independent experiments, and quantitative data are represented as mean ± SD (*n* = 3).

E17 and NA7 mouse antibodies showed specificity for the target receptors in both malignant and non‐tumorigenic breast cell lysates with bands observed for IL‐6R (~50–55 kDa) and GP130 (~130 to 150 kDa) (Figure 3A). IL‐6 treatment induced a statistically significant 3‐fold and 5.5‐fold rise in levels of phospho‐STAT3 (pSTAT3) in MCF‐7 and MDA‐MB‐231 cell lines with murine E17 and NA7 antibody treatment resulting in a statistically significant reduction in pSTAT3 levels (Figure 3B,C). Endothelial tube formation in HUVEC cells in vitro indicated that IL‐6 treatment induced a significant increase in the number of endothelial tubes formed but was inhibited after treatment with the E17 and NA7 mouse antibodies (Figure 3D,E). Thus, based on characterization and functional screening, the E17 anti‐IL‐6R and NA7 anti‐GP130 mouse monoclonal antibody clones were selected for humanization and further functional characterization.

### 3.4 Humanization of the E17 and NA7 Monoclonal Antibodies

**FIGURE 4:**
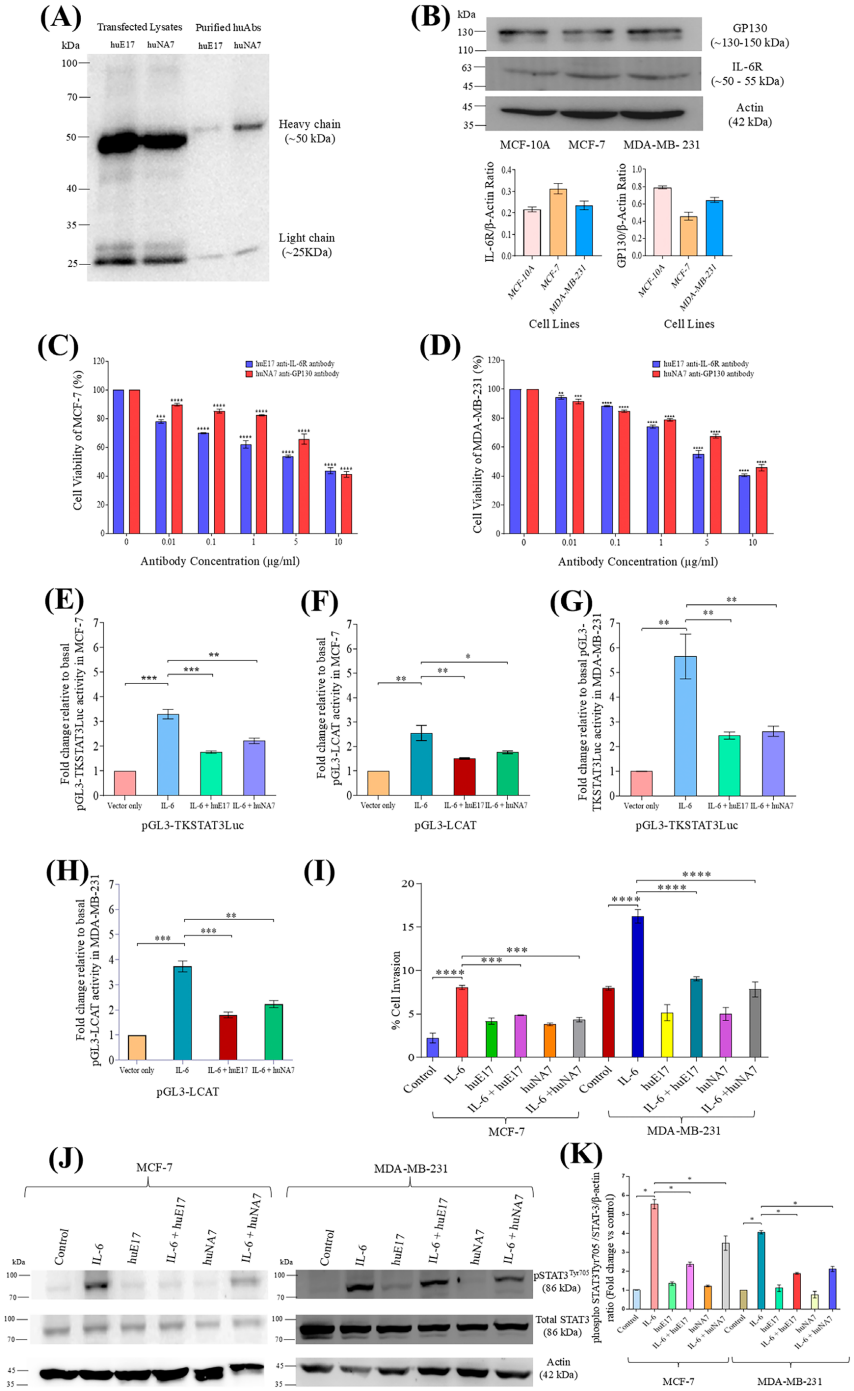
Confirmation of huE17 and huNA7 humanization and in vitro characterization: (A) Confirmation of huE17 and huNA7 humanization (B) IL‐6R and GP130 binding specificity of huE17 and huNA7 antibodies. (C, D) Cytotoxicity of huE17 and huNA7 in MCF‐7 and MDA‐MB‐231 cell lines. Statistical significance for panels C and D was determined using two‐way ANOVA with Dunnett's multiple comparisons test (*p ≤ 0.05, **p ≤ 0.01, ***p ≤ 0.001 and ****p ≤ 0.0001). (E‐H) pGL3‐TKSTAT3Luc response elements luciferase reporter assay and pGL3‐LCAT promoter luciferase reporter vector assay in MCF‐7 and MDA‐MB‐231 cells. (I) Basement membrane assay to assess effect of humanized antibodies on IL‐6 induced invasive ability. Statistical significance for panels E‐I was determined by one‐way ANOVA with Sidak's multiple comparisons test (*p ≤ 0.05, **p ≤ 0.01, ***p ≤ 0.001 and ****p ≤ 0.0001). (J, K) western blot and densitometric assessment of neutralizing effect of huE17 anti‐IL‐6R and huNA7 anti‐GP130 antibodies on IL‐6 induced phosphorylation of STAT3 in breast cancer cell lines. pSTAT3 levels were normalized to Total STAT3 and β‐Actin levels and represented as fold change versus control. Statistical significance for indicated pairwise comparisons was determined by two‐tailed unpaired *t*‐test with Welch's correction (*p ≤ 0.05, **p ≤ 0.01, ***p ≤ 0.001 and ****p ≤ 0.0001). Representative western blots and data from cytotoxicity assays, luciferase reporter assays and basement membrane assay are shown from independent experiments and quantitative data are represented as mean ± SD (*n* = 3).

The humanized E17 and NA7 V_H_ and V_L_ encoding nucleotide sequences were cloned into pVITRO1‐M80‐F2‐IgG1/κ vector. Vector maps for the humanized antibody encoding plasmids are indicated in Figure S6. Bands at the molecular weight range of around 50 kDa and 25 kDa, corresponding to IgG heavy and light chains were observed after western blotting of purified humanized antibodies and transfected cell lysates and probing with anti‐human IgG secondary antibody (Figure 4A). ColabFold generated 3D structures for the huE17 and huNA7 antibodies are indicated in Figure S7A,B. The corresponding 3D structures generated for the Fv regions of huE17 and huNA7 are represented in Figure S8A,B respectively. The highest ranked docking poses for the huE17/IL‐6R and huNA7/GP130 antibody‐receptor interactions from the ClusPro server are represented in Figures S9 and S11 respectively. Computational binding affinity estimation using CSM‐AB yielded negative predicted Gibbs free energy scores for huE17/IL‐6R (Figure S10) and huNA7/GP130 (Figure S12). The putative epitope residues identified for the huE17/IL‐6R and huNA7/GP130 antibody‐receptor complexes are listed in Tables S1 and S2 respectively. Docking‐based epitope mapping suggested that huE17 engages residues across domains II and III of IL‐6R, while the predicted huNA7 epitope on GP130 localized predominantly to domains II and III.

### 3.5 In Vitro Functional Characterization of huE17 and huNA7

The huE17 and huNA7 monoclonal antibodies for IL‐6R and GP130 retained specificity for the targeted receptors in cell lines with bands observed between 50 to 55 kDa and 130 to 150 kDa which correspond with the reported molecular weights for both receptors, while no non‐specific binding was observed (Figure 4B). Both huE17 and huNA7 displayed dose‐dependent effects on cell viability with IC_50_ values of 5 μg/mL (huE17) and 6–7 μg/mL (huNA7) in both cell lines (Figure 4C,D). IL‐6 induced an approximately 3‐fold and 5.5 to 6‐fold increase respectively in luciferase activity levels in MCF‐7 (Figure 4E) and MDA‐MB‐231 (Figure 4G) cells respectively in the STAT3 response reporter assay which was significantly suppressed by huE17 and huNA7. A similar pattern was observed in LCAT promoter activity with the humanized antibodies suppressing IL‐6 induced 2.5‐fold and 3.5‐fold increase in LCAT promoter activity in MCF‐7 (Figure 4F) and MDA‐MB‐231 (Figure 4H) cells. Basement membrane invasion assay demonstrated that huE17 and huNA7 significantly reduced IL‐6 induced invasion, decreasing MCF‐7 invasion from 8% to 4% and MDA‐MB‐231 invasion from 18% to 8% (Figure 4I). Western blotting (Figure 4J) and densitometric analysis (Figure 4K) confirmed robust inhibition of IL‐6–induced STAT3 phosphorylation by both humanized antibodies in both cell lines.

### 3.6 huE17 and huNA7 Modulate IL‐6 Associated Angiogenesis and Tumour Growth In Vivo

**FIGURE 5:**
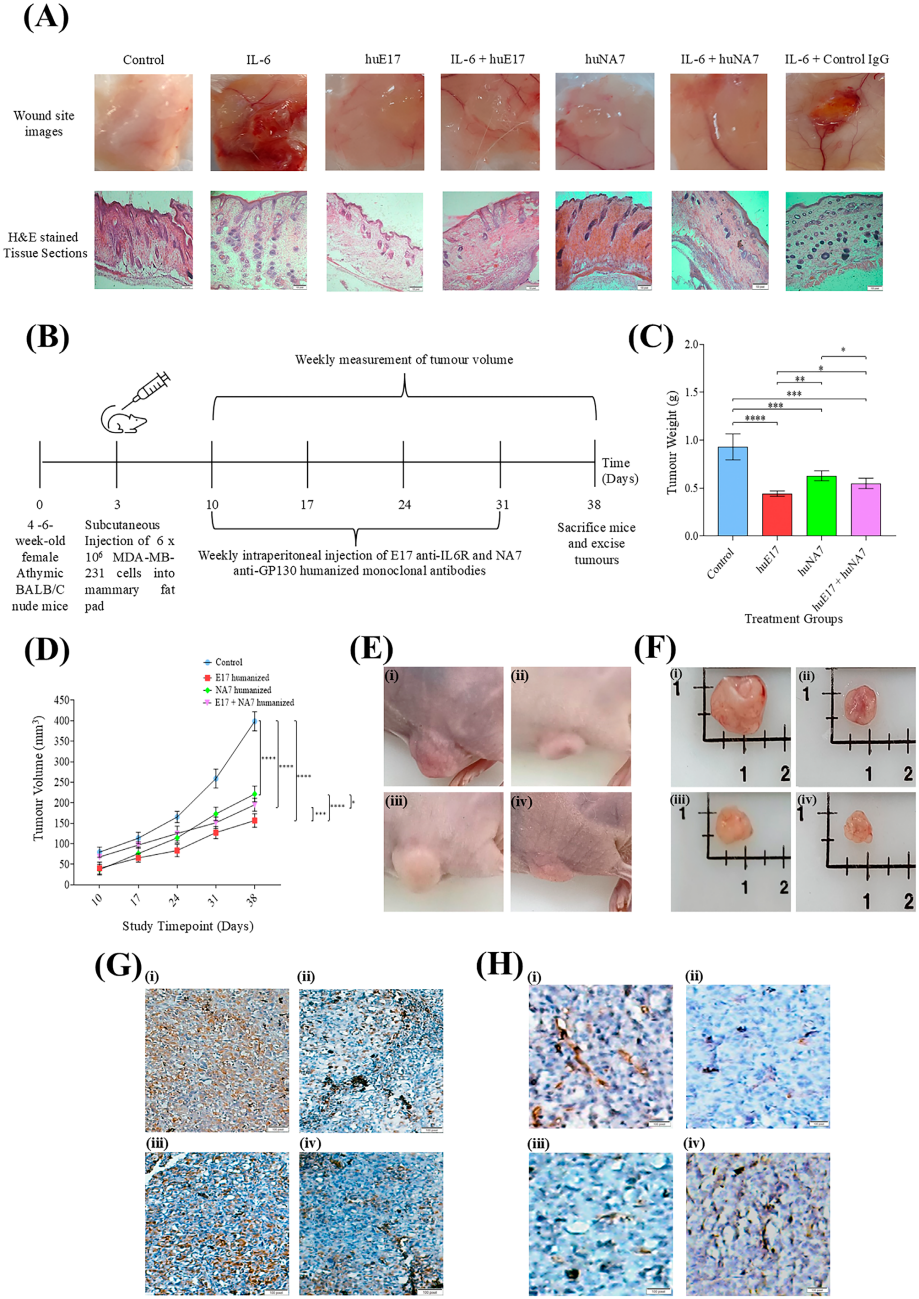
In vivo functional characterization of huE17 and huNA7: (A) Representative wound site images and H&E‐stained sections to assess effect of huE17 and huNA7 antibodies on IL‐6 induced neovascularization in vivo. In vivo angiogenesis assay was performed using *n* = 6 mice/group. Scale bars = 100 μm. (B) Schematic overview of nude mouse orthotopic breast tumour xenograft study. (C) Tumour weight measurement post treatment with huE17, huNA7 or combination therapy. Statistical significance was determined using one‐way ANOVA with Sidak's multiple comparisons test (*p ≤ 0.05, **p ≤ 0.01, ***p ≤ 0.001 and ****p ≤ 0.0001). (D) Effect of humanized antibodies on tumour volume progression. Statistical significance was determined using two‐way ANOVA with Tukey's multiple comparisons test (*p ≤ 0.05, **p ≤ 0.01, ***p ≤ 0.001 and ****p ≤ 0.0001). Quantitative tumour weight and tumour volume data are represented as mean ± SD (*n* = 4 mice/group). (E, F) Representative tumour images before and after tumour excision respectively. (G, H) Representative images of IHC staining for vimentin (10× magnification) and CD31 (20× magnification); (i) control (ii) huE17 antibody (iii) huNA7 antibody (iv) huE17 + huNA7 antibody. Scale bars = 100 μm.

In the agarose plug transplantation assay both gross examination and H&E staining revealed IL‐6 induced increased vessel formation which was abrogated by treatment with huE17 and huNA7 humanized antibodies (Figure 5A). A schematic overview of the orthotopic breast tumour xenografts study in nude mice is outlined in Figure 5B. Individual as well as combined huE17 and huNA7 antibody treatments significantly reduced tumour weights compared to untreated controls (Figure 5C). Combination treatment with huE17 and huNA7 also resulted in a significantly greater reduction in tumour weight compared to huNA7 treatment alone. huE17 treatment resulted in the greatest reduction in tumour volume, while combined huE17 and huNA7 treatment resulted in a greater tumour volume reduction than huNA7 alone (Figure 5D). Representative tumour images were taken both before and after tumour excision and are shown in Figure 5E,F. Immunohistochemistry revealed a trend of higher vimentin expression (Figure 5G) in untreated compared to the antibody treated tumours. CD31 staining revealed reduced number of CD31 positive blood vessels in antibody treated tumours compared to untreated controls which was consistent with the results from the agarose plug transplant assay (Figure 5H).

## 4 Discussion

In this study, we report the development and preclinical characterization of two humanized monoclonal antibodies targeting IL‐6R and GP130 and evaluate their effects on IL‐6 signalling and downstream tumour‐related phenotypes in breast cancer models. Through a combination of in vitro and in vivo assays, we assessed the ability of these antibodies to modulate IL‐6–driven STAT3 activation, tumour cell invasion, angiogenesis and tumour growth.

The murine antibody clones E17 and NA7 were selected based on target specificity, attenuation of IL‐6 induced STAT3 phosphorylation and anti‐angiogenic ability. Humanization through CDR‐grafting eliminated potential immunogenicity, while preserving antigen specificity and functional efficacy. This was supported by molecular modelling and in vitro assessments including cytotoxicity assays, STAT3 and LCAT responsive elements reporter assays, inhibition of IL‐6 induced STAT3 activation and invasion assays. In vivo evaluation demonstrated that both huE17 and huNA7 reduced tumour growth and angiogenesis in orthotopic breast cancer xenograft and agarose plug models. Collectively, these findings support the ability of the developed antibodies targeting IL‐6R or GP130 to disrupt the IL‐6 signalling cascade in preclinical breast cancer models. In vivo, IL‐6R directed targeting with huE17 resulted in the most pronounced reduction in tumour burden, while combined huE17 and huNA7 administration produced tumour growth suppression greater than GP130 targeting alone.

A key aspect of this study is the evaluation of concurrent targeting of IL‐6R and GP130 in breast cancer models. In context of breast cancer, individual inhibition of IL‐6R has been investigated and shown to attenuate IL‐6/STAT3 signalling and tumour progression [54]. In parallel, GP130 has also been targeted with small‐molecule inhibitors such as bazedoxifene demonstrating the ability to suppress tumour growth in preclinical cancer models [34]. However, despite these advances, antibody‐mediated dual targeting of both IL‐6R and GP130 has not been systematically evaluated in breast cancer. By assessing IL‐6R and GP130 blockade individually and in combination using in vivo breast tumour xenografts, our study demonstrates that concurrent receptor targeting is a biologically viable option capable of suppressing IL‐6 associated tumour growth. While IL‐6R directed targeting with huE17 resulted in the most pronounced reduction in tumour burden, the observed tumour growth attenuating ability of combined IL‐6R and GP130 blockade supports further exploration of dual receptor targeting to more comprehensively suppress IL‐6 signalling in breast cancer.

GP130 also functions as a signalling receptor for multiple other IL‐6 family cytokines including OSM, LIF, IL‐11 and IL‐27. Therefore, broad GP130 blockade could potentially affect physiological cytokine signalling [23]. However, IL‐6 signalling cascade driven STAT3 activation in breast cancer is frequently sustained and dysregulated relative to normal tissue homeostasis [16]. Thus, for GP130 blockade to become a viable therapeutic option, controlled dosing regimen, optimized treatment schedules, tumour‐directed delivery strategies, or combination approaches may be needed to minimize unintended systemic cytokine suppression while preserving anti‐tumour efficacy.

The anti‐tumour effects observed post IL‐6R and GP130 blockade may reflect inhibition of IL‐6 trans‐signalling. IL‐6 trans‐signalling enables IL‐6 responsiveness in GP130 expressing tumour cells independent of membrane bound IL‐6R expression by means of soluble IL‐6R mediated signalling, thus facilitating oncogenic signalling even in breast tumours with low or heterogeneous IL‐6R expression. IL‐6 trans‐signalling has been associated with immunosuppression, stemness, enhanced proliferation and migration in breast cancer models [55, 56, 57]. Thus, huE17 and huNA7 may contribute towards comprehensive suppression of IL‐6 driven tumour promoting pathways. In contrast, IL‐6 cluster signalling is poorly characterized in breast cancer and warrants further investigation.

Several therapeutic agents targeting the IL‐6/IL‐6R/GP130 signalling axis are clinically approved for autoimmune and inflammatory diseases [31]. Some of these agents, including tocilizumab, have been assessed as treatment modalities for breast cancer. In vitro and in vivo studies show that tocilizumab blocks IL‐6 induced STAT3 phosphorylation and represses proliferative, migratory, invasive effects and angiogenic effects [54], but regulatory approval has not been achieved. GP130 has been comparatively less explored in cancer, and there are currently no clinically approved humanized anti‐GP130 antibodies for breast cancer. In this context, our findings extend existing knowledge by providing preclinical evidence that antibody‐mediated targeting of GP130, as well as concurrent targeting of both IL‐6R and GP130, can modulate IL‐6–driven tumour phenotypes in breast cancer models.

Residue‐level comparison suggested no direct overlap between the predicted huE17 epitope and the experimentally defined tocilizumab and sarilumab binding interfaces, both of which are reported to localize predominantly to domain III of IL‐6R, which is primarily involved in ligand binding to the receptor complex [58]. Docking‐based epitope mapping suggested that huE17 engages a contiguous binding surface spanning domains II and III of the IL‐6R ectodomain, which would be consistent with interference at the level of receptor architecture rather than solely blockade of the ligand binding. Furthermore, the predicted huNA7 epitope localized primarily to domains II and III of GP130, which are directly involved in cytokine‐induced receptor dimerization. Together these observations support the potential of concurrent receptor targeting as a therapeutic strategy, as inhibition of IL‐6R or GP130 alone may not fully suppress both IL‐6 classical and trans‐signalling [59] and provide a plausible structural basis for the inhibitory activity of these antibodies.

The present study focused on preclinical characterization through in vitro and in vivo systems to establish biological activity of the humanized antibodies. As such direct comparisons with existing therapeutics and detailed pharmacokinetic evaluations were not undertaken. Further validation in patient‐derived and immunocompetent breast cancer models will be important to evaluate the tumour‐immune interactions following IL‐6R and GP130 blockade and strengthen translational relevance. Given the association of elevated IL‐6/STAT3 signalling with poor prognosis, tumour progression and recurrence [23, 24], these antibodies may be particularly relevant to breast cancer patients with elevated IL‐6 levels and heightened cytokine‐driven signalling activity.

In summary, we describe the development and functional characterization of two humanized monoclonal antibodies—huE17 and huNA7 targeting the IL‐6R and GP130 receptors respectively. Both antibodies effectively modulate IL‐6 associated signalling and tumour growth in breast cancer models. By evaluating individual and combined receptor‐complex targeting, this work provides mechanistic and functional insight into IL‐6 pathway targeting for therapy and supports continued exploration of dual IL‐6R and GP130 targeting as a future therapeutic strategy in breast cancer.

## Author Contributions

**Keshava Prasad:** investigation. **Guruprasad Baipadithaya:** methodology, formal analysis, investigation. **Ganesh Prasad Uppenda Gopalakishna:** investigation. **Arun Chandrashekar:** methodology, investigation, resources. **Manjunath B. Joshi:** methodology, formal analysis. **Ritam Naha:** investigation. **Ramyaa Periasamy:** investigation. **Satyajit Dey Pereira:** methodology, investigation, validation, formal analysis, writing – original draft, visualization. **Shama Bhat:** funding acquisition, resources. **Kapaettu Satyamoorthy:** conceptualization, methodology, investigation, formal analysis, writing – review and editing, funding acquisition, supervision. **Lavanya Prakash Acharya:** investigation.

## Funding

The present study was funded by grants from the Department of Biotechnology (DBT)‐Biotechnology Industry Research Assistance Council (BIRAC) under the Biotechnology Industry Partnership Program (BIPP) (Grant No: BT/BIPP05443/17/11) and by the Indian Council of Medical Research (ICMR), Government of India (Grant No. 2019‐3353).

## Ethics Statement

All animal experiments were approved by the Institutional ethics committees at Bhat BioTech India(P) Ltd. (CPSEA No. 1350/C/10/CPCSEA; Study no: BBI/IAEC/JULY/2017/01 and BBI/IAEC/JULY/2018/02) and Kasturba Medical College, Manipal India (CPCSEA no: 94/PO/ReBi/S/1999/CPCSEA; Study no: IAEC/KMC/2019/48) respectively. Umbilical cord samples for HUVEC isolation were obtained after approval from the Institutional ethics committee at Kasturba Medical College, Manipal India with informed consent of patients (Study no: IEC/514/2019).

## Conflicts of Interest

The authors declare no conflicts of interest.

## Supporting information


**Figure S1:** Cloning strategy for receptor interacting domains and hybridoma development. cDNA sequences encoding domain I, II and III of GP130 receptor and domains II and III of IL‐6R receptor were cloned into pOptiVEC TOPO vector between the EcoRI and XhoI (NEB, USA) restriction sites and then transfected into CHO cells, Ni‐NTA resin affinity purification was used to purify recombinant proteins which were then used to immunize BALB/C mice to generate mouse antibodies and subsequently develop hybridomas.
**Figure S2:** Overview of domains along with amino acid coordinates for (A) IL‐6R and (B) GP130 receptors. The ectodomains of the IL‐6R and GP130 receptors that contribute to the formation of the hexameric IL‐6/IL‐6R/GP130 complex are indicated.
**Figure S3:** (A) Visualization through molecular modelling of interaction between IL‐6 (PDB ID:1ALU) and the ectodomains of IL‐6R (PDB ID:1N26) and GP130 (PDB ID: 3L5H) that form the IL‐6/IL‐6R/GP130 Signalling complex. (B) IL‐6R Domain II (Fibronectin Type III 1) and III (Fibronectin Type III 2) (P113 to M311) and GP130 Domain I (Ig like C2 type), Domain II (Fibronectin Type III) and Domain III (Fibronectin Type III containing WXSWS motif) (D26 to Y321) are involved in formation of the IL‐6/IL‐6R/GP130 signalling complex. Key amino acids involved in the interaction are highlighted in black.
**Figure S4:** SDS‐PAGE gel images for cell culture supernatants and Ni‐NTA column purified His‐tagged recombinant (A) IL‐6R and (B) GP130 domain proteins that are involved in the IL‐6/IL‐6R/GP130 signalling complex.
**Figure S5:** Validation through mass spectrometric analysis of expression and purification of recombinant ectodomains of (A) IL‐6R and (B) GP130 that are involved in IL‐6/IL‐6R/GP130 signalling complex.
**Figure S6:** Vector Maps for the humanized antibody encoding (A) pVITRO‐E17 anti‐IL‐6R and (B) pVITRO‐NA7 anti‐GP130 plasmid.
**Figure S7:** Structure prediction of (A) huE17 antibody with the heavy and light chains in pink and green respectively and V_H_ and V_L_ regions highlighted in red and blue respectively and the CDR regions overlaid with white markings. (B) huNA7 antibody with the heavy and light chains in yellow and purple and V_H_ and V_L_ regions marked in orange and red respectively with CDR regions overlaid with white markings.
**Figure S8:** Molecular modelling of predicted structures of the Fv regions of (A) Humanized E17 anti‐IL‐6R antibody with the V_H_ and V_L_ regions marked in red and yellow respectively and the CDR regions overlaid with white and cyan markings respectively and (B) Humanized NA7 anti‐GP130 antibody with the V_H_ and V_L_ regions marked in magenta and cyan respectively and the CDR regions overlaid with yellow and orange markings respectively.
**Figure S9:** Molecular docking of the predicted Fv region of huE17 with IL‐6R receptor ectodomains (PDB ID: 1N26): The highest ranked predicted antibody receptor complex obtained through molecular modelling carried out using the ClusPro server along with docking score is represented.
**Figure S10:** Binding affinity estimation (Predicted delta G) between huE17 predicted Fv region structure and IL‐6R ectodomains using the CSM‐AB tool.
**Figure S11:** Molecular docking of the predicted Fv region of huNA7 with GP130 receptor ectodomains (PDB ID: 3L5H). The highest ranked predicted antibody receptor complex obtained through molecular modelling carried out using the ClusPro server along with docking scores is represented.
**Figure S12:** Binding affinity estimation (Predicted delta G) between huNA7 predicted Fv region structure and GP130 ectodomains using the CSM‐AB tool.
**Table S1:** Putative IL‐6R epitope residues identified from docking‐based analysis of the huE17 antibody predicted Fv region structure with IL‐6R receptor ectodomains (PDB ID: 1N26).
**Table S2:** Putative GP130 epitope residues identified from docking‐based analysis of the huNA7 antibody predicted Fv region structure with GP130 receptor ectodomains (PDB ID: 3L5H).
**Supplementary Methods**. Generation of constructs with IL‐6 responsive elements to produce reporter systems and humanization of murine E17 anti‐IL‐6R and NA7 anti‐GP130 antibodies.

## Data Availability

The data that support the findings of this study are available from the corresponding authors upon reasonable request.
