# Supplementary material for: Humanized Antibodies Targeting Ectodomains of IL‐6R and GP130 Suppress IL‐6/STAT3 Signalling and Tumour Growth in Breast Cancer Models
Source: J Cell Mol Med. 2026 Jun 18;30(12):e71250. doi: 10.1111/jcmm.71250 (PMC13279542; doi:10.1111/jcmm.71250)
Supplement: Supplementary file 1 — Figure S1: Cloning strategy for receptor interacting domains and hybridoma development. cDNA sequences encoding domain I, II and III of GP130 receptor and domains II and III of IL‐6R receptor were cloned into pOptiVEC TOPO vector between the EcoRI and XhoI (NEB, USA) restriction sites and then transfected into CHO cells, Ni‐NTA resin affinity purification was used to purify recombinant proteins which were then used to immunize BALB/C mice to generate mouse antibodies and subsequently develop hybridomas. Figure S2: Overview of domains along with amino acid coordinates for (A) IL‐6R and (B) GP130 receptors. The ectodomains of the IL‐6R and GP130 receptors that contribute to the formation of the hexameric IL‐6/IL‐6R/GP130 complex are indicated. Figure S3: (A) Visualization through molecular modelling of interaction between IL‐6 (PDB ID:1ALU) and the ectodomains of IL‐6R (PDB ID:1N26) and GP130 (PDB ID: 3L5H) that form the IL‐6/IL‐6R/GP130 Signalling complex. (B) IL‐6R Domain II (Fibronectin Type III 1) and III (Fibronectin Type III 2) (P113 to M311) and GP130 Domain I (Ig like C2 type), Domain II (Fibronectin Type III) and Domain III (Fibronectin Type III containing WXSWS motif) (D26 to Y321) are involved in formation of the IL‐6/IL‐6R/GP130 signalling complex. Key amino acids involved in the interaction are highlighted in black. Figure S4: SDS‐PAGE gel images for cell culture supernatants and Ni‐NTA column purified His‐tagged recombinant (A) IL‐6R and (B) GP130 domain proteins that are involved in the IL‐6/IL‐6R/GP130 signalling complex. Figure S5: Validation through mass spectrometric analysis of expression and purification of recombinant ectodomains of (A) IL‐6R and (B) GP130 that are involved in IL‐6/IL‐6R/GP130 signalling complex. Figure S6: Vector Maps for the humanized antibody encoding (A) pVITRO‐E17 anti‐IL‐6R and (B) pVITRO‐NA7 anti‐GP130 plasmid. Figure S7: Structure prediction of (A) huE17 antibody with the heavy and light chains in pink and green respectiv [file JCMM-30-e71250-s001.docx]

**Humanized antibodies targeting ectodomains of IL-6R and GP130 suppress IL-6/STAT3 signaling and tumor growth in breast cancer models**

Satyajit Dey Pereira^1*^, Guruprasad Baipadithaya^1^, Keshava Prasad^1^, Ritam Naha^1,a^, Lavanya Prakash Acharya^1,b^, Ganesh Prasad Uppenda Gopalakishna^2^, Ramyaa Perisamy^2^, Arun Chandrashekar^2^, Shama Bhat^2^, Manjunath B. Joshi^3^, Kapaettu Satyamoorthy^4*^

^1^ Department of Cell and Molecular Biology, Manipal School of Life Sciences, Manipal Academy of Higher Education, Manipal, India

^2^ Bhat Bio-Tech India (P) Ltd., Bengaluru, India

^3^ Department of Ageing Research, Manipal School of Life Sciences, Manipal Academy of Higher Education, Manipal, India

^4^ SDM Centre for Cellular and Molecular Sciences, SDM College of Medical Sciences and Hospital, Shri Dharmasthala Manjunatheshwara (SDM) University, Dharwad, India

^a^Present address: Molecular Targets Program, Center for Cancer Research (CCR), National Cancer Institute (NCI), National Institutes of Health (NIH), Frederick, USA

^b^Present address: Institute of Endotypes in Oncology, Metabolism and Immunology (IEOMI), National Research Council (CNR), Naples, Italy

* Corresponding authors

Satyajit Dey Pereira

Department of Cell and Molecular Biology

Manipal School of Life Sciences

Manipal Academy of Higher Education

Manipal, India

Email : Satyajit.pereira@learner.manipal.edu

Dr. Kapaettu Satyamoorthy

Professor

SDM College of Medical Sciences and Hospital,

Sri Dharmasthala Manjunatheshwara (SDM) University,

Dharwad, India

Email: ksatyamoorthy@sdmuniversity.edu.in

**Supplemental Materials**

**Supplementary Methods :**

**Generation of constructs with IL-6 responsive elements to produce reporter systems: Cloning of STAT3 construct into TK-LUC vector:**

STAT3 construct was produced by annealing the following set of oligonucleotides in annealing buffer (10 mM Tris-HCl, pH 7.5, 50 mM NaCl, 1 mM EDTA) at 95℃ for minutes and thereafter at cooling down to room temperature for approximately 30 minutes. The annealed constructs were phosphorylated using T4 Polynucleotide Kinase following the protocol as directed by NEB.

STAT3 Forward: 5’ CTATGCATA**TTCC**TGTAAGTGGGTAC 3’

STAT3 responsive consensus site

STAT3 Reverse: 5’ CCACTTACAGGAATATGCATAGGTAC 3’

The STAT3 construct thus generated would have 3’ sticky end overhangs post digestion with KpnI and could be ligated into the pGL3-TK-Luc vector after digesting the vector with KpnI enzyme. The ligation mixture would be transformed into *E.coli* Dh5α and colonies obtained after transformation were screened for positive clones. The vector with at least 4 STAT construct repeats were selected and validated by sanger sequencing and subsequently used for transfection into different cell lines to study the IL-6 responsiveness through luciferase assay.

**Humanization of murine E17 anti-IL-6R and NA7 anti-GP130 antibodies :**

RNA was isolated by Trizol method from the mouse E17/NA7 antibody producing murine IgG2b hybridoma cells and cDNA conversion carried out. PCR screening was then carried out of the cDNA from the murine hybridomas using different light chain and heavy chain degenerate primer combinations as previously described [36] . Subsequently sanger sequencing of the 500bp amplicons was carried out to obtain the antibody V_H_ and V_L_ nucleotide sequences. Humanization of the E17/NA7 mouse monoclonal antibodies was performed through CDR-grafting as previously described[37].The frame regions (FRs) and complementarity determining regions (CDRs) 1-3 of the V_H_ and V_L_ nucleotide sequences for the E17 and NA7 antibodies were identified using the IMGT/VQUEST database[38]. V_H_ and V_L_ sequences were used as input for TABHU tool which identified a framework donor sequence with high similarity to the mouse monoclonal antibody sequence, grafted the mouse antibody CDR sequences into this sequence, and corrected individual back mutations to generate a humanized antibody sequence with similar binding mode to the mouse antibodies[39]. The V_H_ and V_L_ nucleotide sequences obtained were cloned into the pVITRO1-M80-F2-IgG1/κ plasmids[40] (Invivogen, USA) to generate humanized huE17 anti-IL-6R and huNA7 anti-GP130 antibody encoding pVITRO plasmids. The V_L_ chain sequence was cloned between BspEI and AvrII (NEB, USA) and V_H_ chain sequence between NotI and BsmBI (NEB, USA) restriction sites. The pVITRO humanized antibody plasmids then were transfected into HEK293 cells using lipofectamine 3000 (Invitrogen, USA). Selection of transfected colonies was carried out by treatment with hygromycin (20 µg/ml) (HiMedia, India).

**Supplementary Figures**


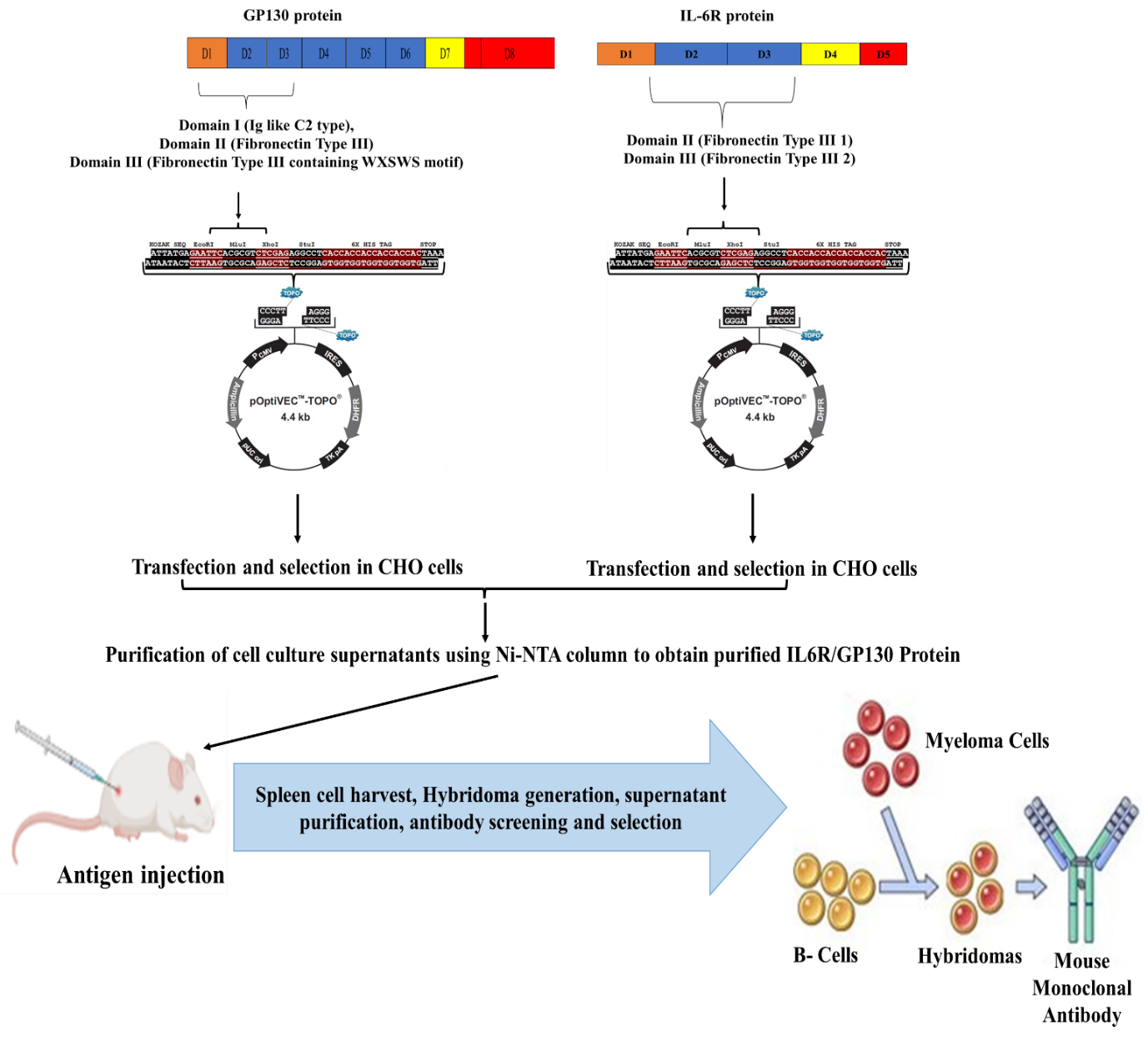


**Supplementary Figure S1 :** Cloning strategy for receptor interacting domains and hybridoma development. cDNA sequences encoding domain I,II and III of GP130 receptor and domains II and III of IL-6R receptor were cloned into pOptiVEC TOPO vector between the EcoRI and XhoI (NEB, USA) restriction sites and then transfected into CHO cells, Ni-NTA resin affinity purification was used to purify recombinant proteins which were then used to immunize BALB/C mice to generate mouse antibodies and subsequently develop hybridomas.

**(A)**


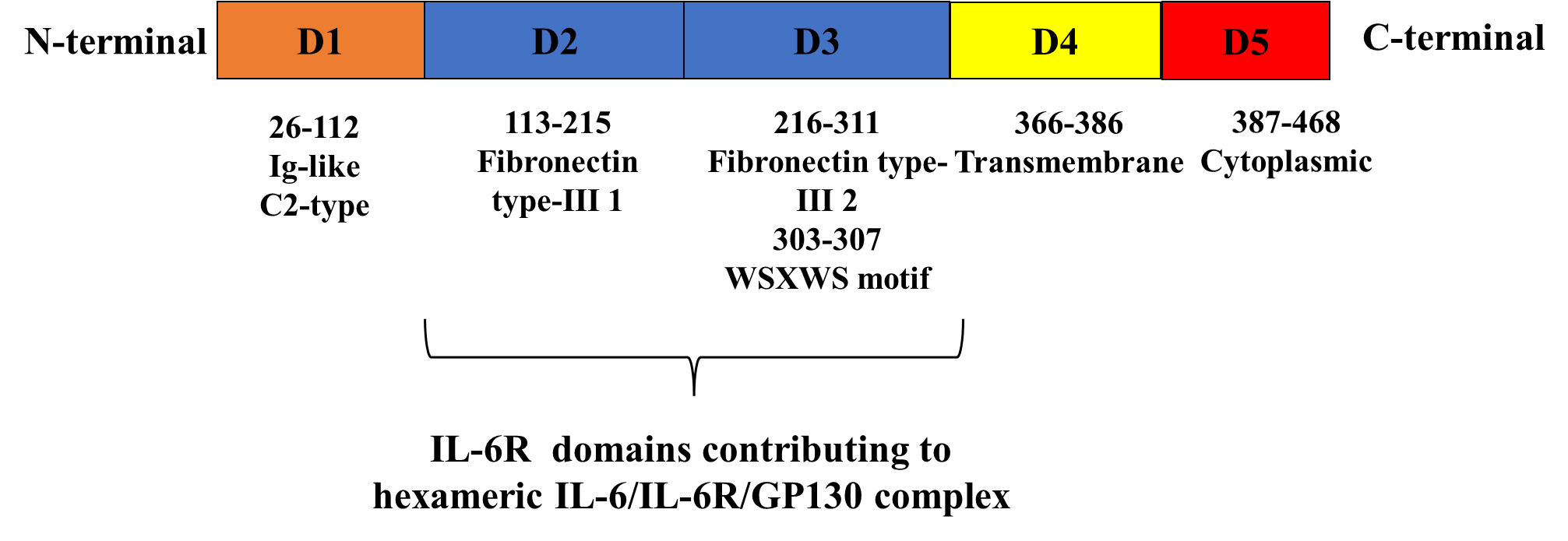


**(B)**


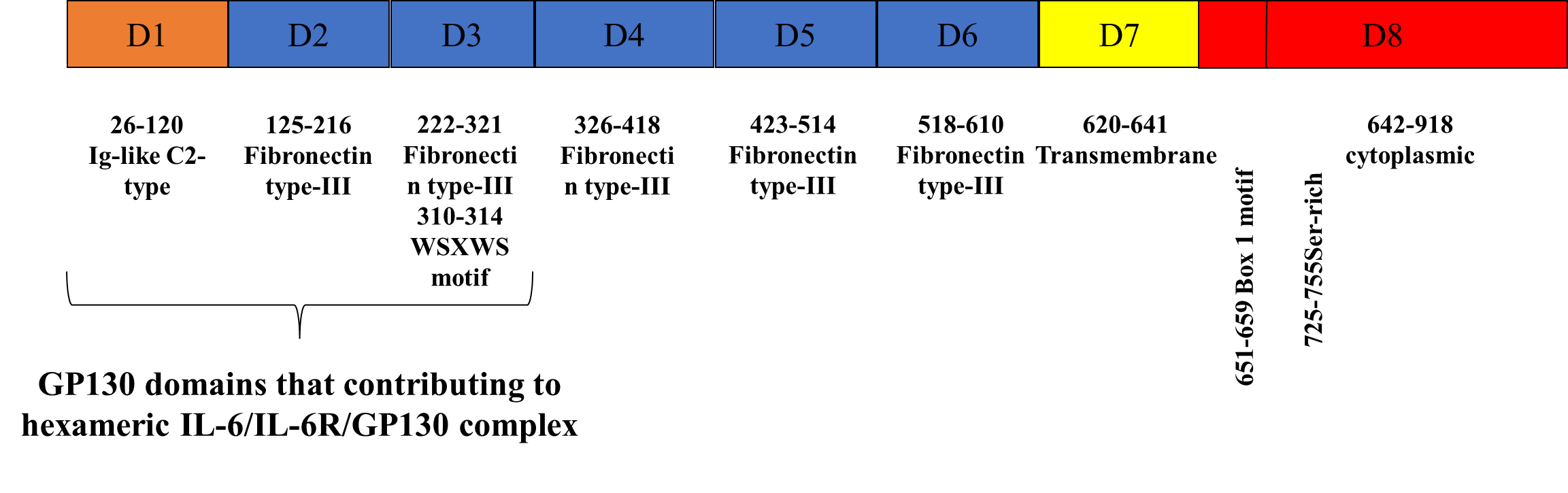


**Supplementary Figure S2 :** Overview of domains along with amino acid coordinates for (A) IL-6R and (B) GP130 receptors. The ectodomains of the IL-6R and GP130 receptors that contribute to the formation of the hexameric IL-6/IL-6R/GP130 complex are indicated.

**(A)**

IL-6


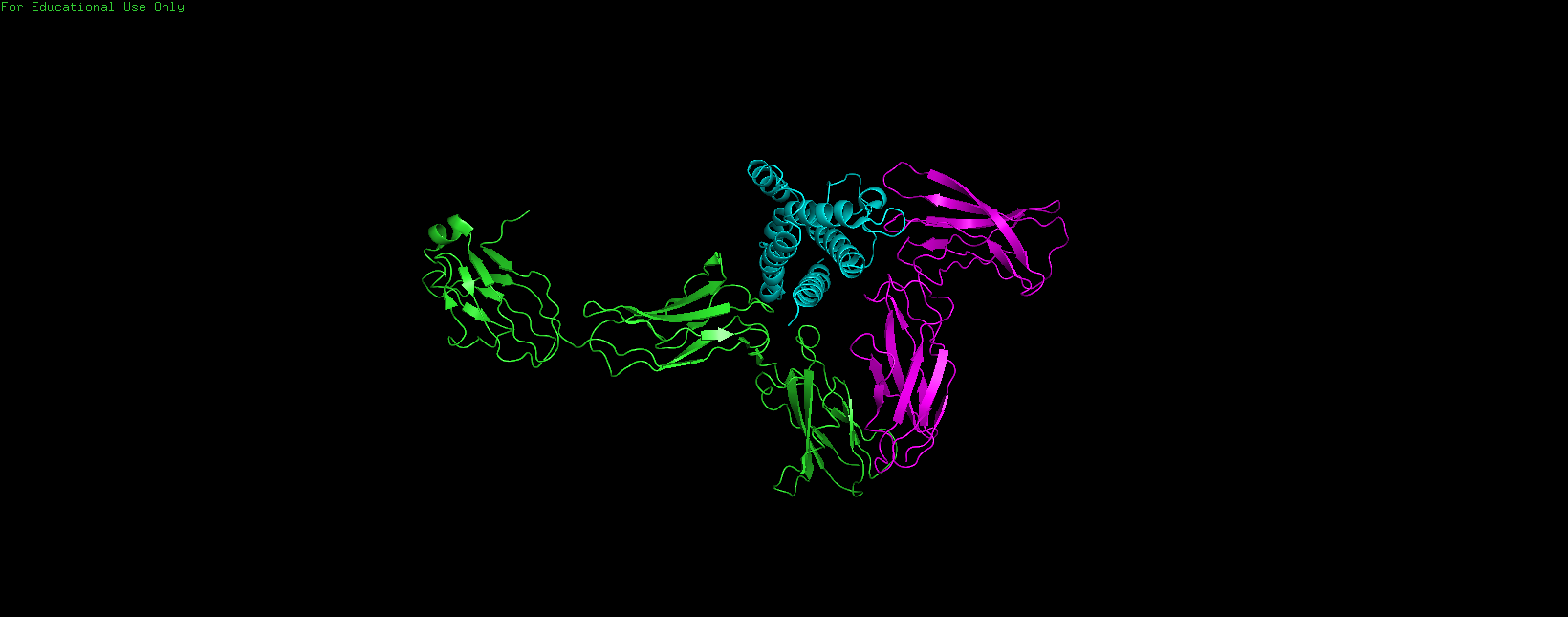


GP130 Domain I & II

IL-6R Domain II

IL-6R Domain III

GP130 Domain III

**(B)**


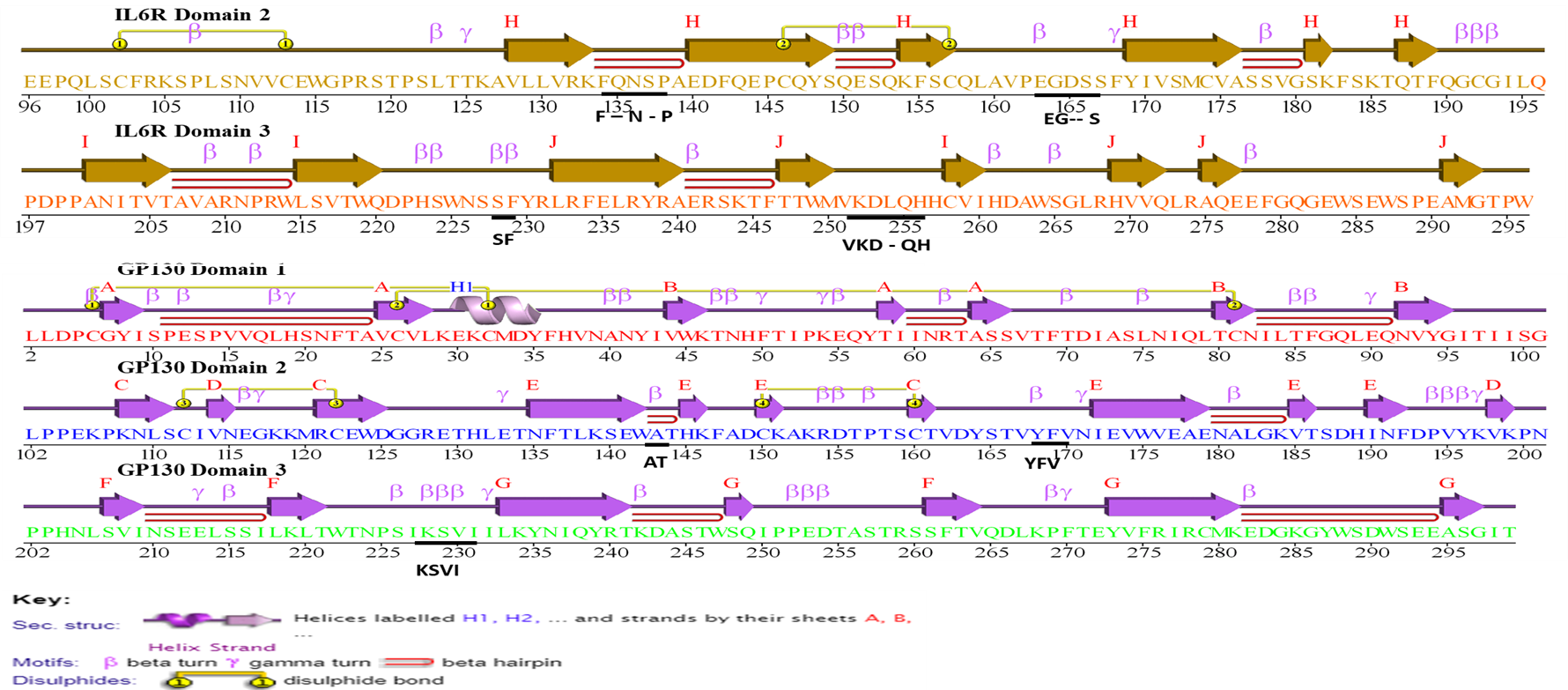


**Supplementary Figure S3 :** (A) Visualization through molecular modelling of interaction between IL-6 (PDB ID:1ALU) and the ectodomains of IL-6R (PDB ID:1N26) and GP130 (PDB ID: 3L5H) that form the IL-6/IL-6R/GP130 Signalling complex. (B) IL-6R Domain II (Fibronectin Type III 1) and III (Fibronectin Type III 2) (P113 to M311) and GP130 Domain I (Ig like C2 type), Domain II (Fibronectin Type III) and Domain III (Fibronectin Type III containing WXSWS motif) (D26 to Y321) are involved in formation of the IL-6/IL-6R/GP130 signalling complex. Key amino acids involved in the interaction are highlighted in black.

**(A) (B)**

1 2 3 4 5 6 7

1 2 3 4 5

| Lane | Description | Lane | Description |
| --- | --- | --- | --- |
| 1 | Protein Marker | 1 | Protein Marker |
| 2 | IL-6R-optivec plasmid transfected CHO cell supernatant | 2 - 4 | GP130-optivec plasmid transfected CHO cell supernatant |
| 3 | IL-6R-optivec plasmid transfected CHO cell supernatant | 5-7 | Ni-NTA affinity chromatography purified recombinant GP130 (approx. 28 kDa) |
| 4 | Ni-NTA affinity chromatography purified recombinant IL-6R (approx. 23 kDa) |  |  |
| 5 | Ni-NTA affinity chromatography purified recombinant IL-6R (approx. 23 kDa) |  |  |

**
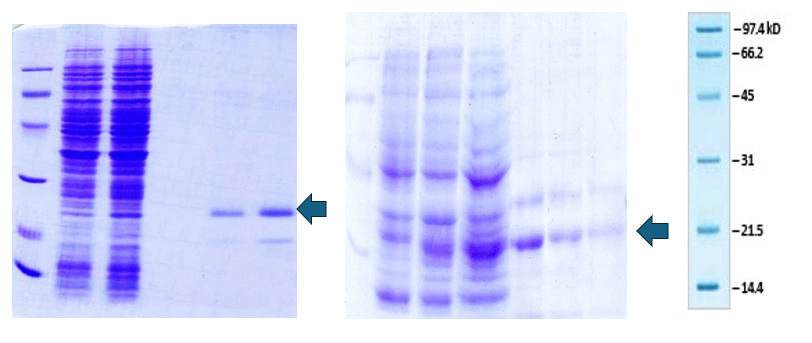
**

**Supplementary Figure S4 :** SDS-PAGE gel images for cell culture supernatants and Ni-NTA column purified His-tagged recombinant (A) IL-6R and (B) GP130 domain proteins that are involved in the IL-6/IL-6R/GP130 signalling complex.

**(b)**

**(a)**

**
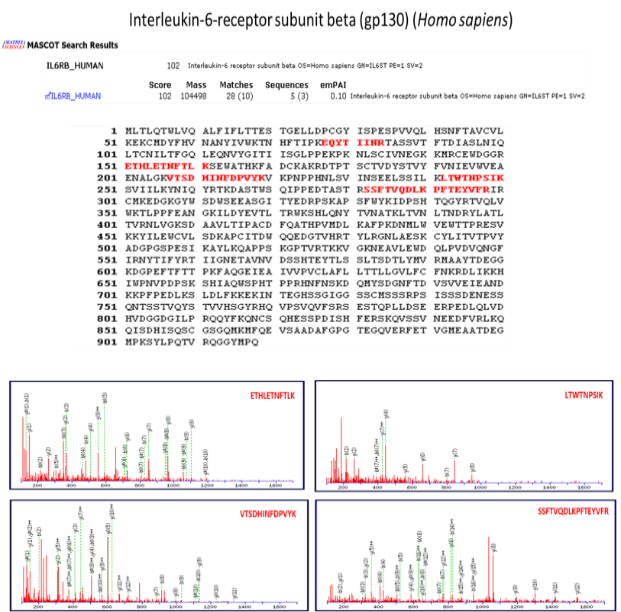

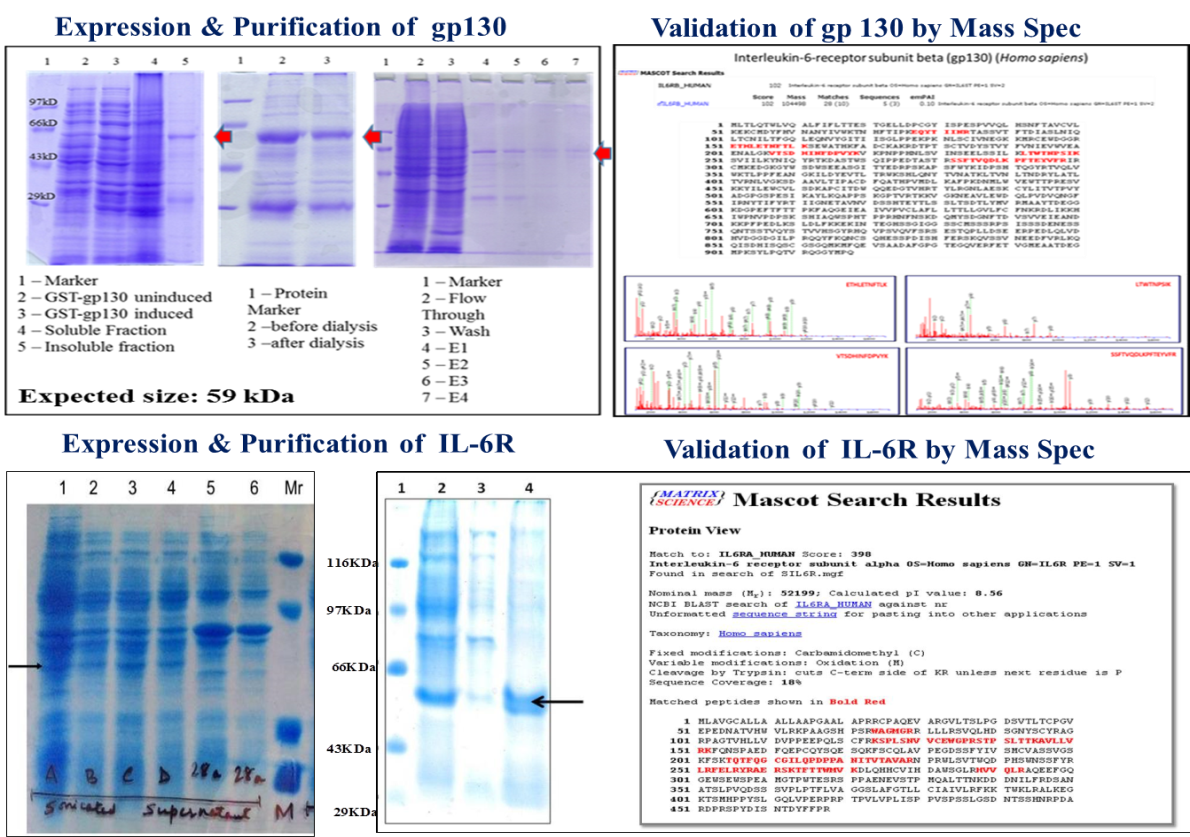
**

**Supplementary Figure S5:** Validation through mass spectrometric analysis of expression and purification of recombinant ectodomains of (A) IL-6R and (B) GP130 that are involved in IL-6/IL-6R/GP130 signalling complex.

**
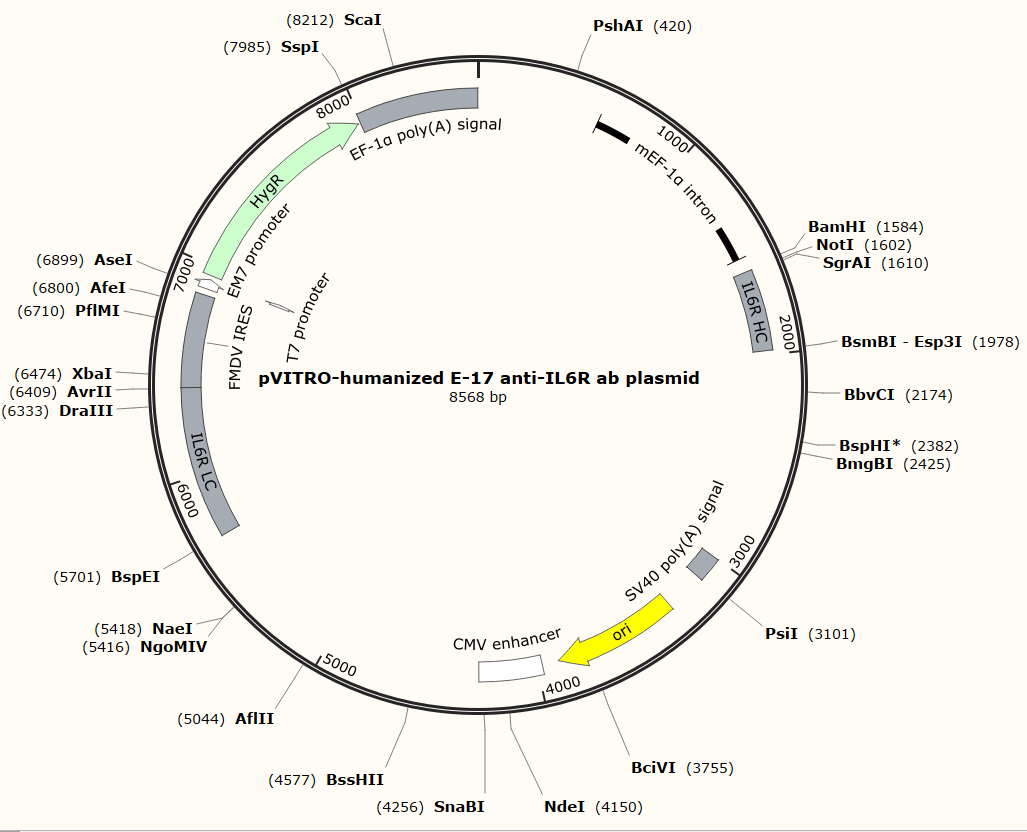
**

**(A)**

**
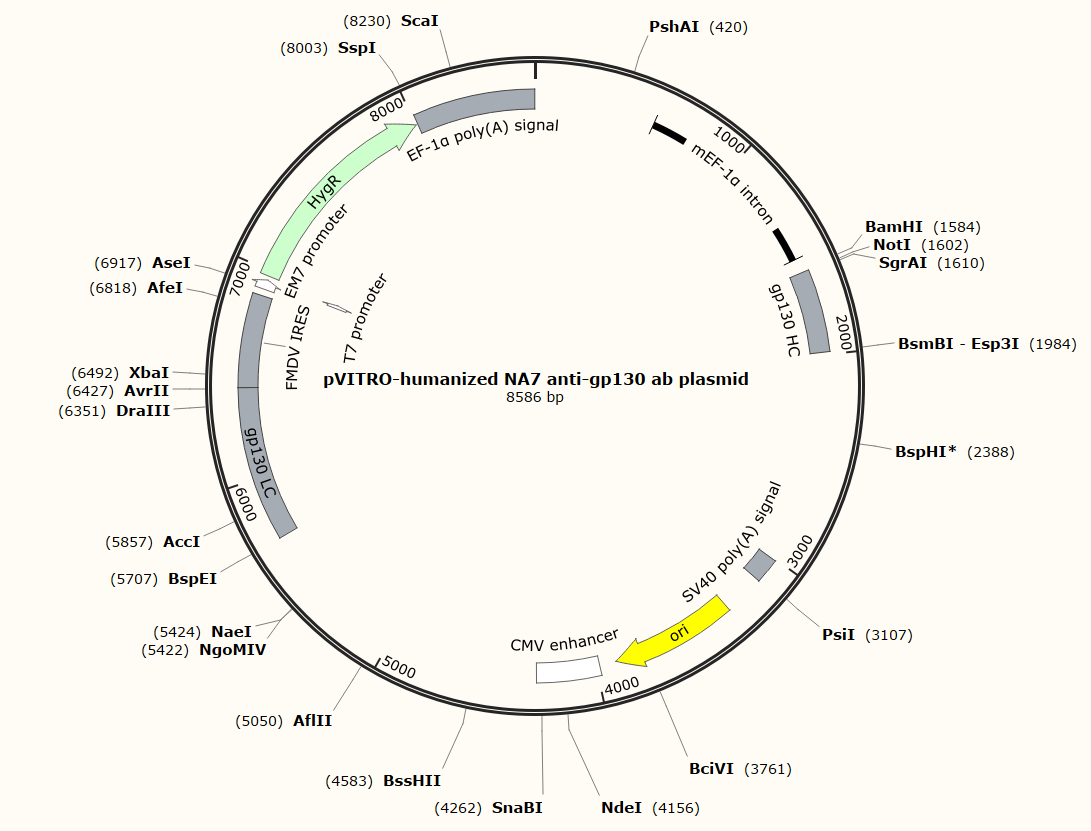
**

**(B)**

**Supplementary Figure S6:** Vector Maps for the humanized antibody encoding (A) pVITRO-E17 anti-IL-6R and (B) pVITRO-NA7 anti-GP130 plasmid.

**(A)**


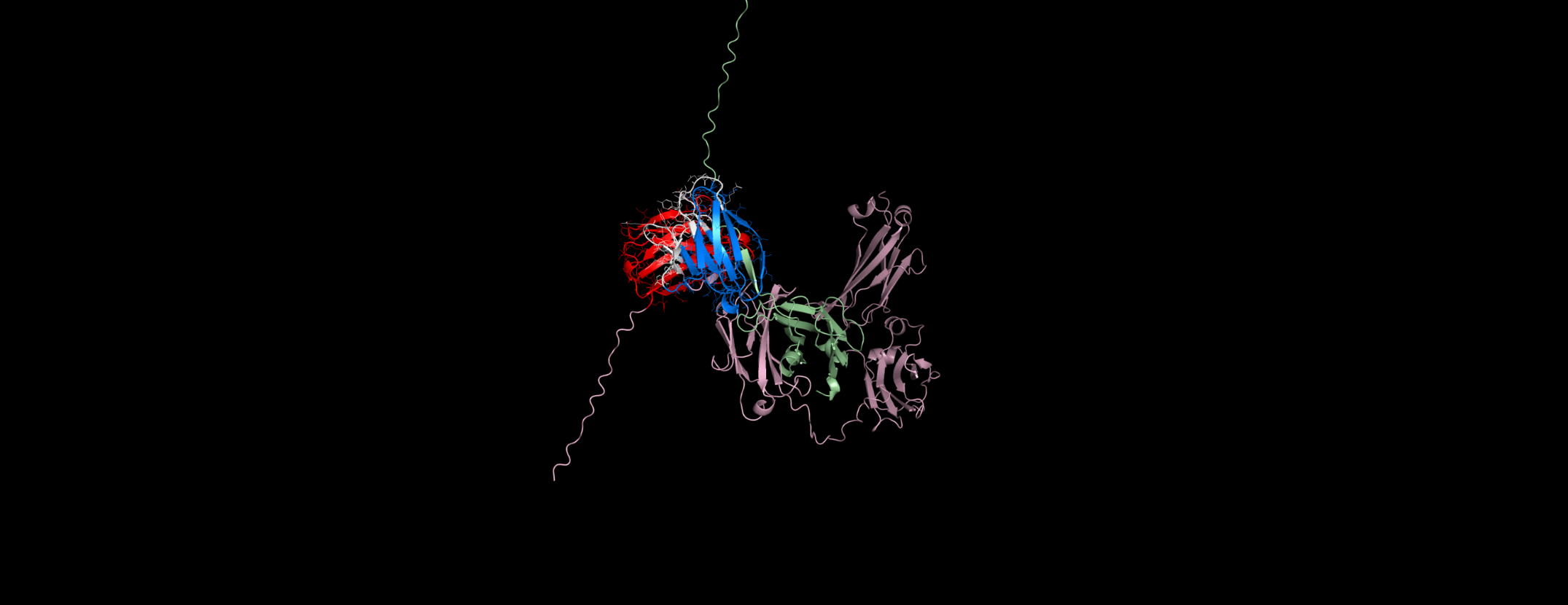


**(B)**


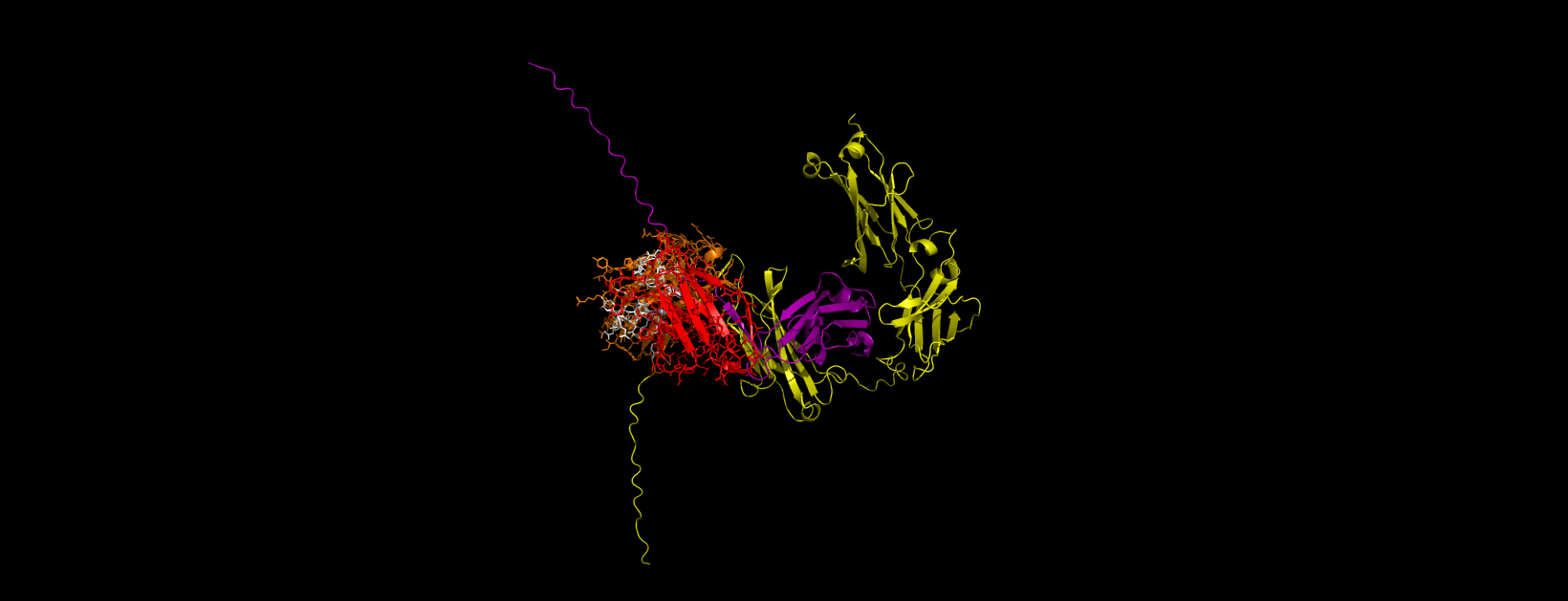


**Supplementary Figure S7:** Structure prediction of (A) huE17 antibody with the heavy and light chains in pink and green respectively and V_H_ and V_L_ regions highlighted in red and blue respectively and the CDR regions overlaid with white markings. (B) huNA7 antibody with the heavy and light chains in yellow and purple and V_H_ and V_L_  regions marked in orange and red respectively with CDR regions overlaid with white markings.

**(A)**

**
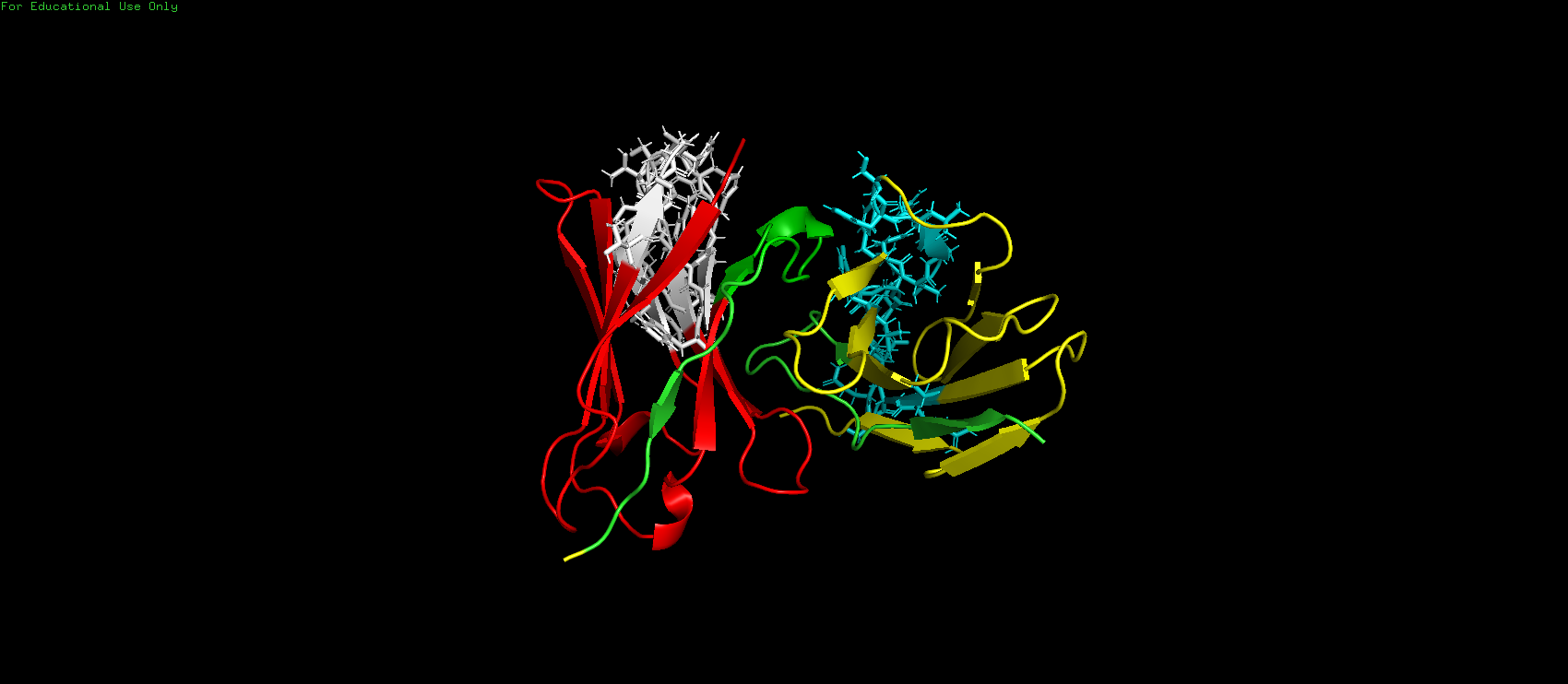
**

**(B)**

**
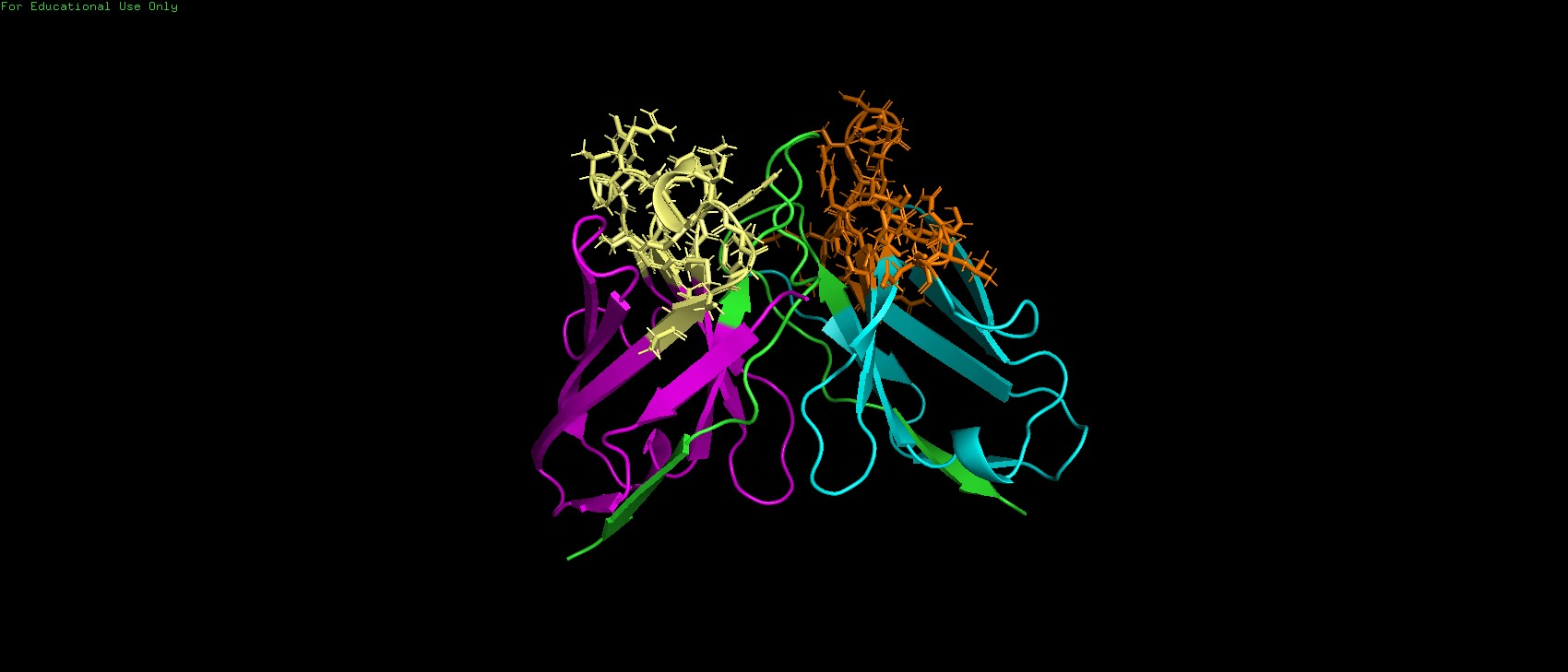
**

**Supplementary Figure S8** : Molecular modelling of predicted structures of the Fv regions of (A) Humanized E17 anti-IL-6R antibody with the V_H_ and V_L_  regions marked in red and yellow respectively and the CDR regions overlaid with white and cyan markings respectively and (B) Humanized NA7 anti-GP130 antibody with the V_H_ and V_L_  regions marked in magenta and cyan respectively and the CDR regions overlaid with yellow and orange markings respectively.


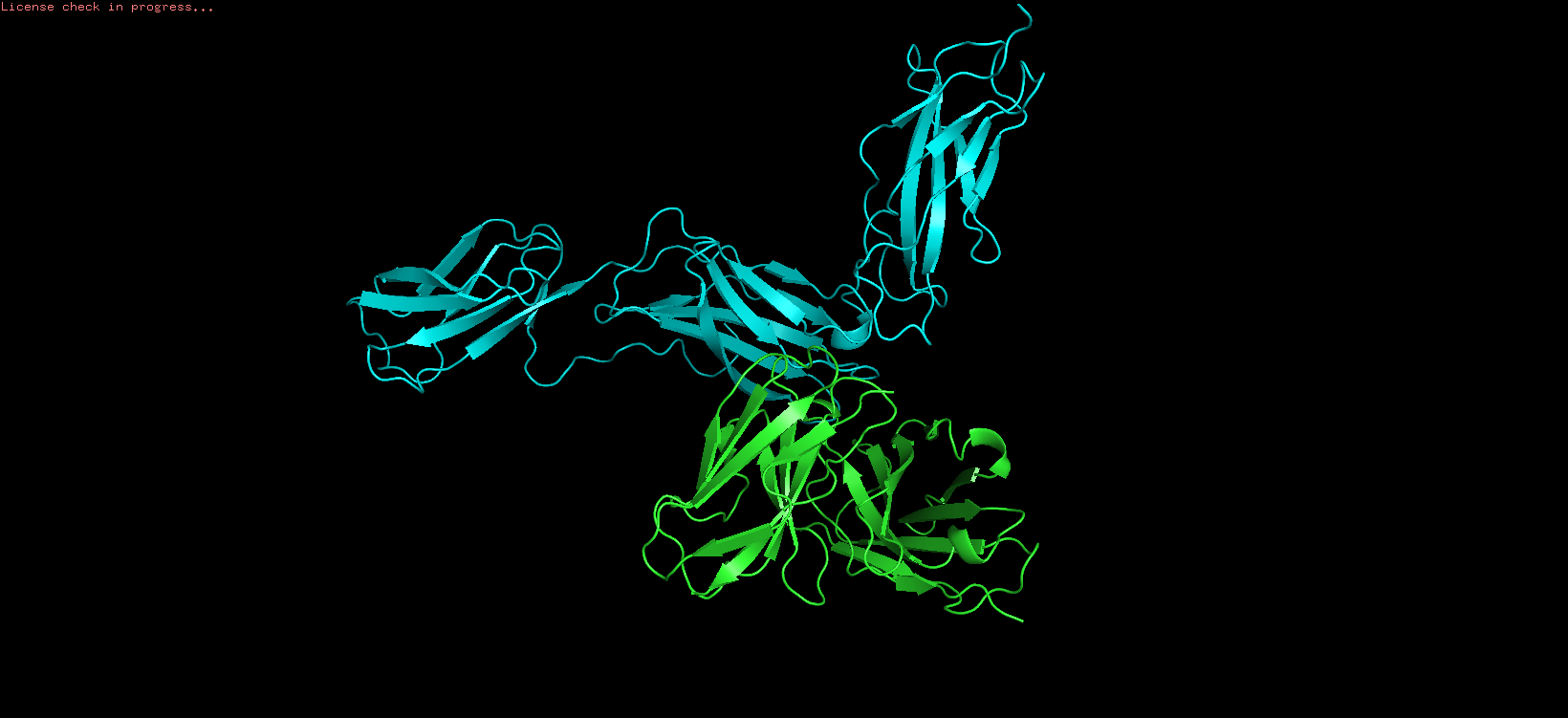


**
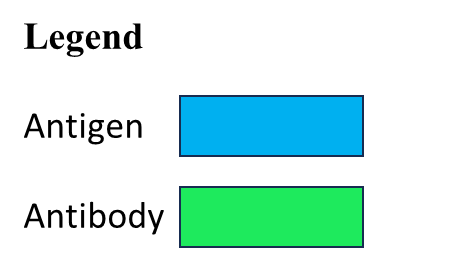
**

| Cluster | Members | Representative | Weighted Score |
| --- | --- | --- | --- |
| 0 | 64 | Center | -252.6 |
| 0 | 64 | Lowest Energy | -264.2 |

**Supplementary Figure S9:** Molecular docking of the predicted Fv region of huE17 with IL-6R receptor ectodomains (PDB ID: 1N26 ) : The highest ranked predicted antibody receptor complex obtained through molecular modelling carried out using the ClusPro server along with docking score is represented.

**
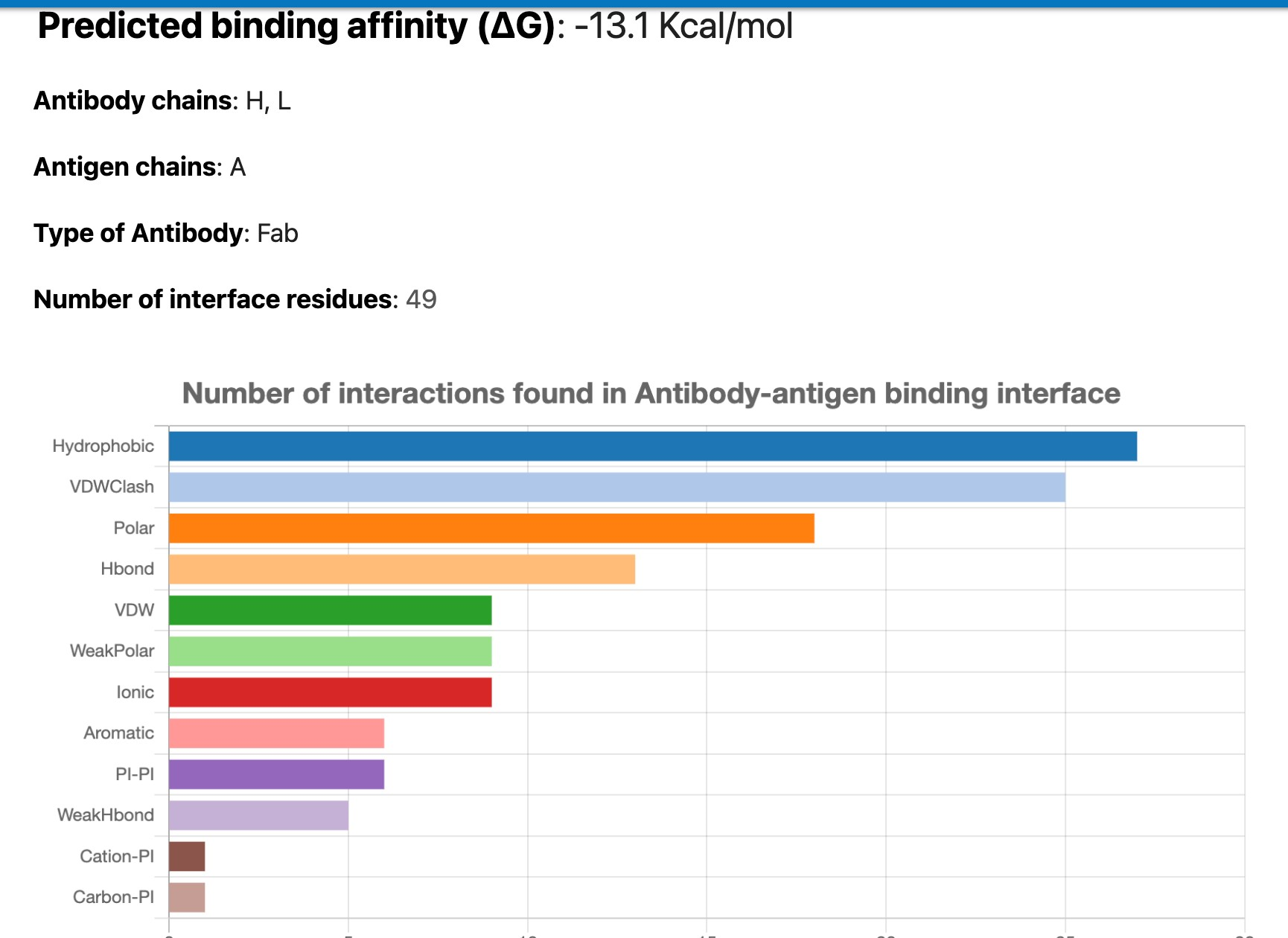
**

**Supplementary Figure S10:** Binding affinity estimation (Predicted delta G) between huE17 predicted Fv region structure and IL-6R ectodomains using the CSM-AB tool.


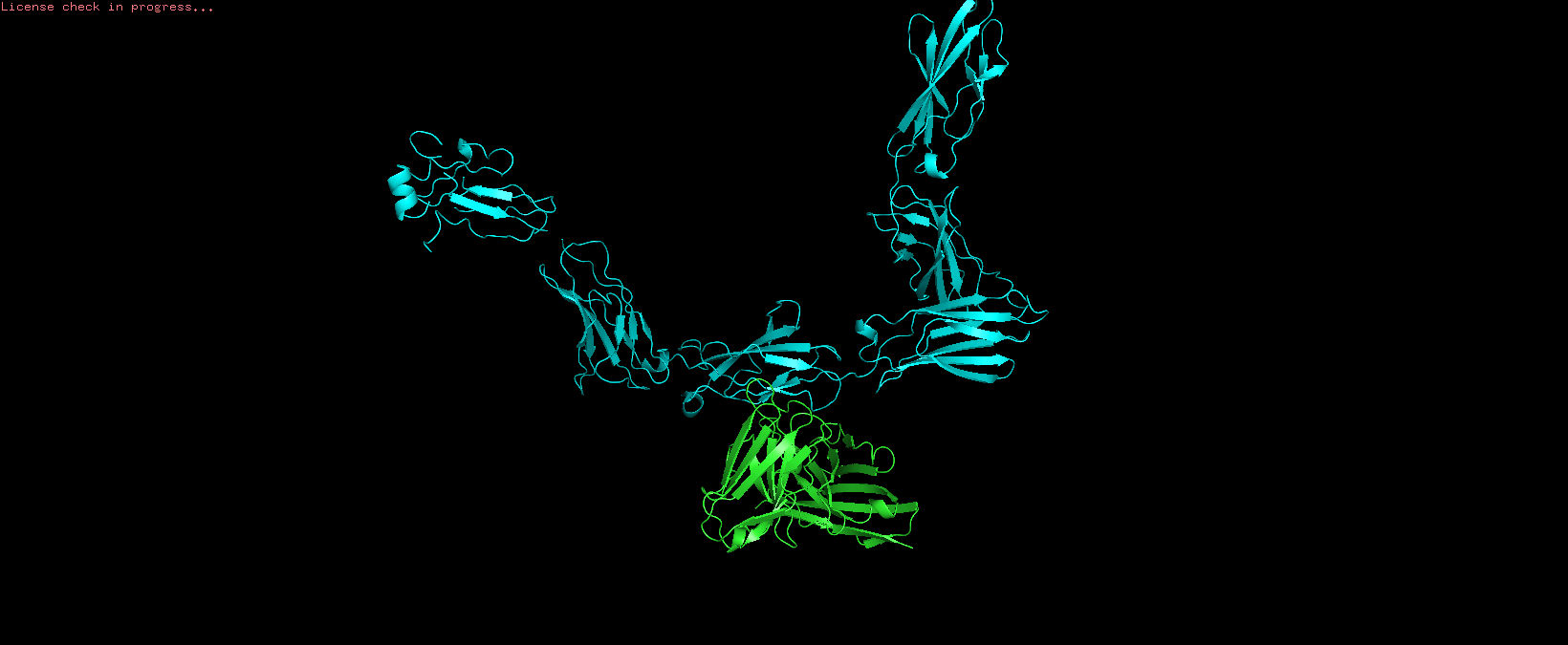


**
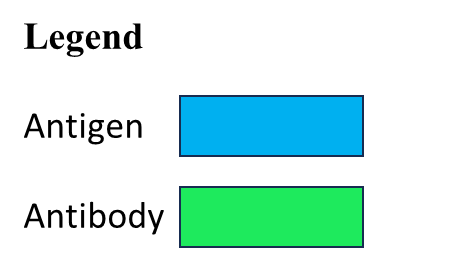
**

| Cluster | Members | Representative | Weighted Score |
| --- | --- | --- | --- |
| 0 | 70 | Center | -338.6 |
| 0 | 70 | Lowest Energy | -338.6 |

**Supplementary Figure S11:** Molecular docking of the predicted Fv region of huNA7 with GP130 receptor ectodomains (PDB ID: 3L5H). The highest ranked predicted antibody receptor complex obtained through molecular modelling carried out using the ClusPro server along with docking scores is represented.

**
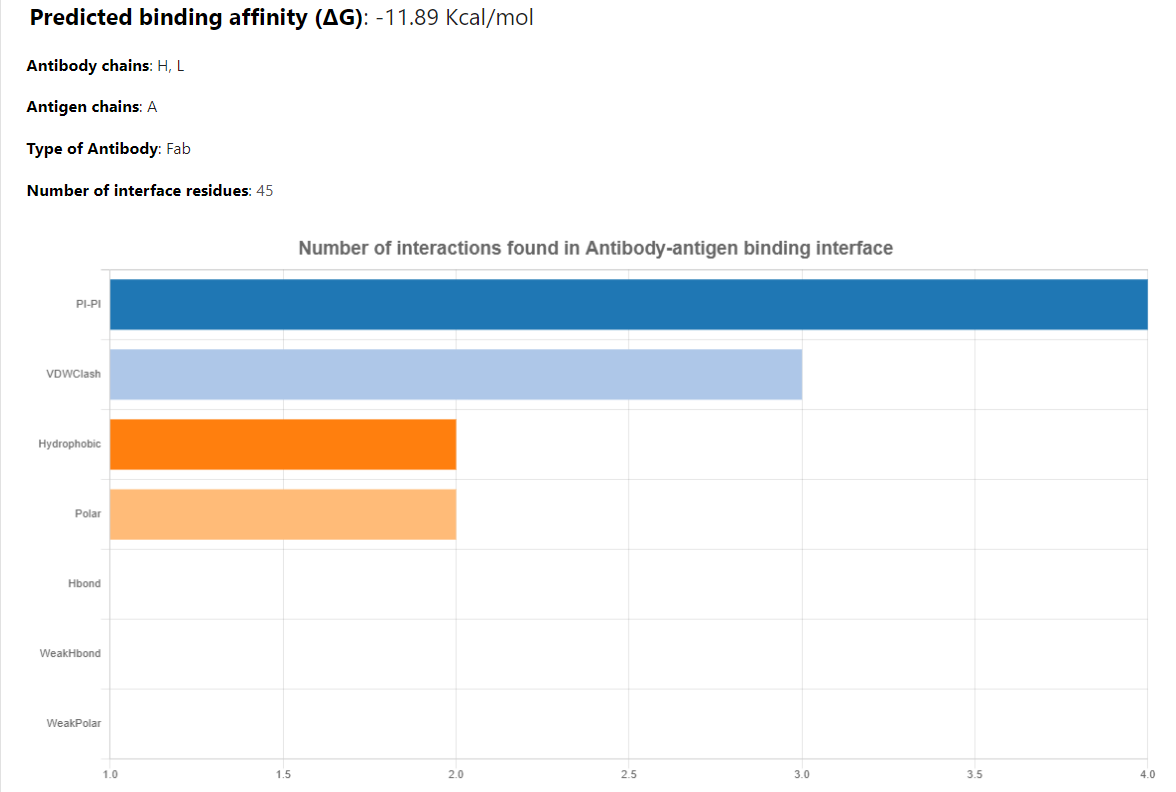
**

**Supplementary Figure S12:** Binding affinity estimation (Predicted delta G) between huNA7 predicted Fv region structure and GP130 ectodomains using the CSM-AB tool.

**Supplementary Tables**

| Serial No. | Residue Position | 3-letter |
| --- | --- | --- |
| 1 | 133 | LYS |
| 2 | 135 | GLN |
| 3 | 136 | ASN |
| 4 | 137 | SER |
| 5 | 140 | GLU |
| 6 | 142 | PHE |
| 7 | 143 | GLN |
| 8 | 144 | GLU |
| 9 | 158 | GLN |
| 10 | 159 | LEU |
| 11 | 160 | ALA |
| 12 | 161 | VAL |
| 13 | 162 | PRO |
| 14 | 165 | ASP |
| 15 | 169 | TYR |
| 16 | 226 | ASN |
| 17 | 227 | SER |
| 18 | 229 | PHE |

**Supplementary Table S1** **:** Putative IL-6R epitope residues identified from docking-based analysis of the huE17 antibody predicted Fv region structure with IL-6R receptor ectodomains (PDB ID : 1N26).

| Serial No. | Residue position | Residue (3-letter) |
| --- | --- | --- |
| 1 | 205 | ASN |
| 2 | 206 | LEU |
| 3 | 207 | SER |
| 4 | 208 | VAL |
| 5 | 209 | ILE |
| 6 | 211 | SER |
| 7 | 212 | GLU |
| 8 | 213 | GLU |
| 9 | 214 | LEU |
| 10 | 219 | LYS |
| 11 | 221 | THR |
| 12 | 223 | THR |
| 13 | 260 | SER |
| 14 | 261 | SER |
| 15 | 293 | GLU |
| 16 | 295 | ALA |
| 17 | 296 | SER |

**Supplementary Table S2 :** Putative GP130 epitope residues identified from docking-based analysis of the huNA7 antibody predicted Fv region structure with GP130 receptor ectodomains (PDB ID : 3L5H).
